# Solution-Processable Redox-Active Polymers of Intrinsic Microporosity for Electrochemical Energy Storage

**DOI:** 10.1021/jacs.2c07575

**Published:** 2022-09-08

**Authors:** Anqi Wang, Rui Tan, Charlotte Breakwell, Xiaochu Wei, Zhiyu Fan, Chunchun Ye, Richard Malpass-Evans, Tao Liu, Martijn A. Zwijnenburg, Kim E. Jelfs, Neil B. McKeown, Jun Chen, Qilei Song

**Affiliations:** † Department of Chemical Engineering, Imperial College London, London SW7 2AZ, U.K.; ‡ Department of Chemistry, Molecular Sciences Research Hub, Imperial College London, London W12 0BZ, U.K.; § EaStChem School of Chemistry, University of Edinburgh, Edinburgh EH9 3FJ, U.K.; ∥ Shanghai Key Laboratory of Chemical Assessment and Sustainability, Department of Chemistry, Tongji University, Shanghai 200092, China; ⊥ Department of Chemistry, University College London, London WC1H 0AJ, U.K.; # Key Laboratory of Advanced Energy Materials Chemistry (Ministry of Education), Renewable Energy Conversion and Storage Center (RECAST), College of Chemistry, Nankai University, Tianjin 300071, China

## Abstract

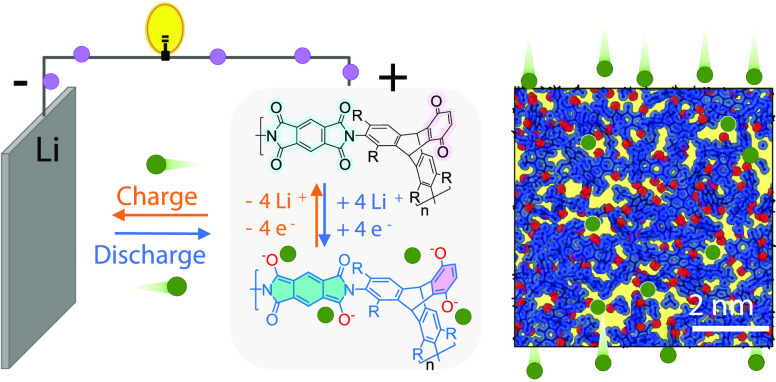

Redox-active organic materials have emerged as promising alternatives to conventional inorganic electrode materials in electrochemical devices for energy storage. However, the deployment of redox-active organic materials in practical lithium-ion battery devices is hindered by their undesired solubility in electrolyte solvents, sluggish charge transfer and mass transport, as well as processing complexity. Here, we report a new molecular engineering approach to prepare redox-active polymers of intrinsic microporosity (PIMs) that possess an open network of subnanometer pores and abundant accessible carbonyl-based redox sites for fast lithium-ion transport and storage. Redox-active PIMs can be solution-processed into thin films and polymer–carbon composites with a homogeneously dispersed microstructure while remaining insoluble in electrolyte solvents. Solution-processed redox-active PIM electrodes demonstrate improved cycling performance in lithium-ion batteries with no apparent capacity decay. Redox-active PIMs with combined properties of intrinsic microporosity, reversible redox activity, and solution processability may have broad utility in a variety of electrochemical devices for energy storage, sensors, and electronic applications.

## 1 Introduction

With the rapid development of renewable energy and electric vehicles, there are increasing demands for efficient and cost-effective batteries for grid storage and transport applications. Lithium-ion batteries (LIBs) dominate the market of portable electronics and electric vehicles with electrode materials mainly produced from inorganic metal oxides, such as LiCoO_2_, LiMn_2_O_4_, LiFePO_4_, and LiNi_*x*_Mn_*y*_Co_*z*_O_2_. However, there are growing concerns about these inorganic electrode materials regarding their high cost, heavy metal toxicity, environmental pollution, and sophisticated end-of-life recycling. In contrast, redox-active organic materials that can reversibly store and release charges have emerged as promising alternatives to inorganic metal oxides owing to their various inherent merits such as chemical diversity, structural tunability, processability, environmental sustainability, and potential low cost.^1,2^ Inspired by the ubiquitous redox cofactors (e.g., quinone- and flavin-based species) in biological systems, the discovery and development of redox-active organic materials have attracted considerable interest for use in batteries and supercapacitors, photo-/electrocatalysis, and sensors. Redox-active organic molecules often have a high density of redox sites per mass, which provides high capacity when used as electrode materials in batteries, such as carbonyl-based small molecules, lithium rhodizoate^3^ (580 mAh g^–1^) and cyclohexanehexone^4^ (902 mAh g^–1^). Nevertheless, these molecular species tend to dissolve in electrolyte solvents, such as carbonates and ethers, leading to rapid performance degradation and a short lifetime.^1^ To avoid this dissolution problem, redox-active segments are directly polymerized or incorporated as pendant groups into polymer chains/networks to afford insoluble solids.^5−8^ However, these redox-active polymers often pack efficiently in a dense amorphous solid state, so that ion transport and electron transfer are slower relative to their molecular analogues, resulting in ineffective utilization of redox sites within the materials and poor electrochemical performance at the device level.

The recent development of porous organic materials provides extended chemical space for designing redox-active organic materials with improved electrochemical performance. The presence of molecular-sized channels constitutes effective pathways for efficient ion diffusion and mass transport, making redox sites more accessible to metal ions and analytes.^9,10^ For example, covalent organic frameworks (COFs) have been extensively studied as electrode materials in supercapacitors and metal-ion batteries,^11−13^ and show rapid redox processes owing to their ordered ion-transport channels, enabling high-power batteries.^14,15^ However, complicated processing is often required to fabricate these crystalline materials into battery electrodes to provide good dispersion and contact between active materials and carbon additives, which are crucial for mixed ionic-electronic conduction.^16^ Several fabrication methods have been reported, such as exfoliation of COF solids into nanosheets,^17^ growth of conductive polymers within COF pores,^18^ and in situ growth of COF nanosheets on carbon nanotubes^12,14,15^ and graphene.^19,20^ These possessing steps add additional complexity and cost to the fabrication process and can be difficult to reproduce or scale up. Hence, it is highly desired to develop redox-active microporous polymers that can be dissolved in certain organic solvents for ease of processing but remain insoluble in electrolytes.

Polymers of intrinsic microporosity (PIMs) are solution-processable microporous materials with subnanometer channels generated from the inefficient packing of the rigid, contorted polymer chains.^21,22^ PIMs have shown great promise as next-generation membrane materials for molecular separations,^23−25^ batteries,^26−29^ and fuel cells.^30^ We anticipate that if incorporated with redox-active structural units, PIMs with fast ion-transport channels could be used as high-performance electrode materials in electrochemical devices. However, the electrochemical properties of PIMs have been rarely investigated, with the exception of using PIM-7 as a membrane separator in Li-S batteries.^31^ We surveyed the library of reported PIMs and noticed four examples that were initially designed for gas separation applications but contain a redox-active structural unit, namely the phenazine in PIM-7^32^ and TPIM-1^33^ as well as the conjugated diimide in PMDA-TMDAT and NTDA-TMDAT^34^ (Figure S2). However, a large portion of redox-inactive structural units is essential in these PIMs to enforce a rigid, contorted chain structure, resulting in a low theoretical charge capacity with values in the range of 90–130 mAh g^–1^. The relatively low density of redox sites represents an obstacle to the immediate use of these PIMs in electrochemical devices. Although an alternative approach toward electroactive PIMs can be achieved via vacuum thermolysis that produces microporous carbon electrodes for supercapacitor energy storage,^35^ the intrinsic merits of PIMs (e.g., solution processability) are sacrificed during the postsynthetic treatment. Therefore, a molecular redesign is required to maximize the mass ratio of redox-active units within the rigid, contorted polymer chains to provide a new generation of solution-processable redox-active PIMs.

**Figure 1:**
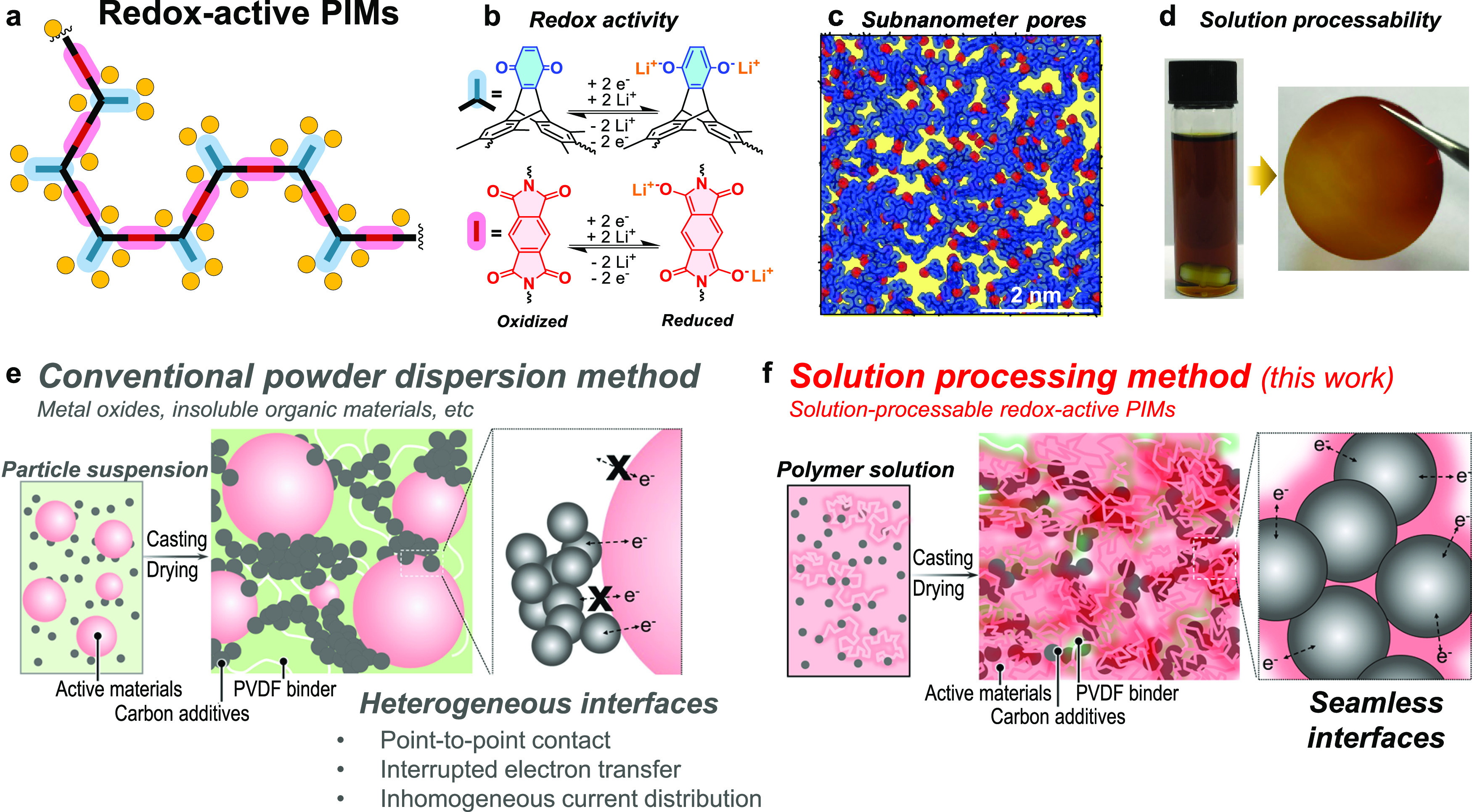
Redox-active polymers of intrinsic microporosity (PIMs). Redox-active PIMs show combined features of reversible redox activity, microporosity generated from inefficient packing of polymer chains for electrolyte uptake and fast ion transport, as well as solution processability for ease of fabrication. (a) Schematic diagram showing the rigid, contorted structure of PIMs. The yellow spheres are metal ions. (b) Redox reaction mechanism of the structural units and linking groups of redox-active PIMs. (c) Structure of a 1 nm thick cross-section of the 49.8 × 49.8 × 49.8 Å^3^ amorphous cells of PIM-TMTrip-MQ with van der Waals surface of the polymer chains shaded in blue and free volume shaded in yellow. Redox-active oxygen atoms are highlighted in red. (d) Photos of a PIM-TMTrip-MHQ NMP solution and a solution-cast self-supported film with a diameter of 2.4 cm. (e, f) Schematic diagrams showing the different microstructures of active material–carbon composites fabricated via the conventional powder dispersion method (e) and the solution possessing method (f).

Here, we report the design and synthesis of redox-active PIMs and demonstrate their promising electrochemical performance in lithium-ion batteries (Figure 1). The material design is achieved by replacing one benzo group of the triptycene component with a benzoquinone unit to afford a redox-active structural unit that remains highly rigid and contorted (Figure 1b). To suppress rotational freedom around the C–N single bond of the imide unit, methyl groups are introduced to exert sufficient steric hindrance. Redox-active PIMs give a high apparent BET surface area of up to ∼700 m^2^ g^–1^ and a high theoretical capacity of up to 248 mAh g^–1^. Moreover, Li^+^ ion diffusion is enhanced by the highly interconnected subnanometer pores of redox-active PIMs (Figure 1c), as verified by cyclic voltammetry (CV) and electrochemical impedance spectroscopy (EIS) measurements, while solution processability allows the fabrication of thin films (Figure 1d) and polymer–carbon composites with a well-defined microstructure (Figure 1f), enabling highly stable cycling performance of lithium-ion batteries. Conventional battery electrodes are usually prepared by a powder dispersion method, i.e., dispersing active materials (usually micron-sized), carbon additives, and polymer binders in a solvent to form a slurry for casting. The resulting electrodes have multiple heterointerfaces between each component, generating high interfacial resistance and unstable microstructures that tend to degrade during repeated charging and discharging (Figure 1e). In contrast, solution-processable redox-active PIMs afford a well-defined electrode microstructure, where active materials are uniformly coated onto carbon additives creating seamless interfaces, which will benefit ion transport and electron transfer processes and promote a homogeneous current density distribution within the electrodes (Figure 1f). The solution processing approach demonstrated in this work for electrode microstructure optimization leads to a remarkable enhancement of cycling stability of lithium-ion batteries and may be applied to other electrochemical devices that require intimate contact between multiple components.

## 2 Results and Discussion

### 2.1 Design, Synthesis, and Characterization of Redox-Active PIMs

Cycloimidization reaction was chosen as the polymerization route to redox-active PIMs as the imide linking group possesses robust redox chemistry^36^ and can be directly generated by careful selection of dianhydride monomers, a conjugated structure of which is required to give reversible redox activity. As the rigid, planar pyromellitic dianhydride (PMDA) was selected for polymerization, a rigid diamine monomer featuring a site of contortion is required to avoid an intractable linear polymer. Based on a previous battery electrode work in the literature, a triptycene quinone molecule was selected as the structural unit as it had previously shown multielectron redox reactions as high-capacity cathode material.^37^ Although its redox cycling performance was reported to be poor due to its partial solubility in battery electrolytes, we anticipated that utilization of this structural unit in redox-active PIMs would simultaneously serve as sites of contortion while providing abundant redox-active functionality.

**Figure 2:**
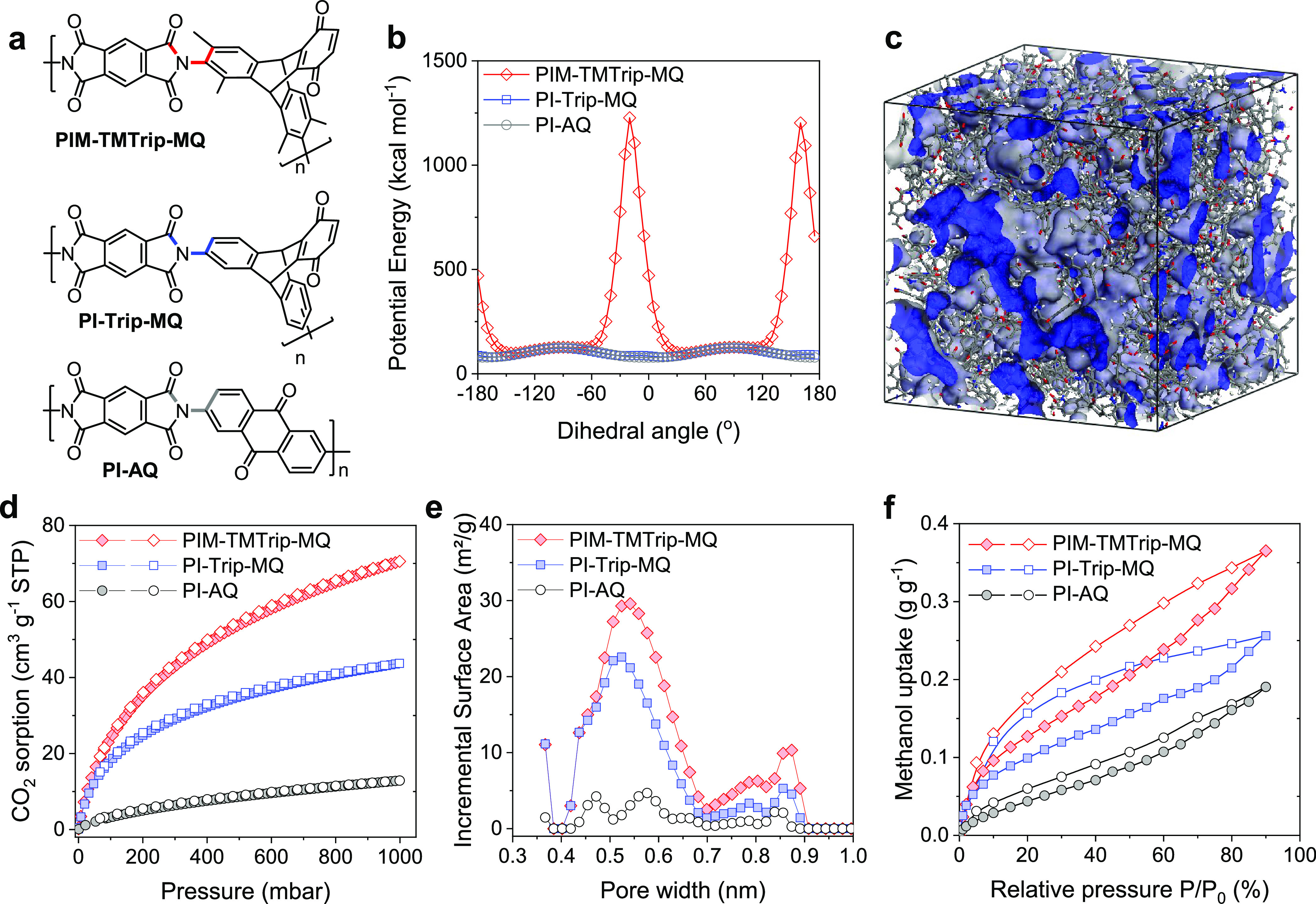
Polymer design and characterization. (a) Chemical structure of redox-active PIMs. (b) Plot showing the increase in energy associated with the deviation in the marked dihedral angle within the imide linking group of PIM-TMTrip-MQ (red) as compared with those in PI-Trip-MQ (blue) and PI-AQ (gray). (c) Three-dimensional view of an amorphous cell of the PIM-TMTrip-MQ polymer. Cell size: 49.8 × 49.8 × 49.8 Å^3^. The blue surface mesh highlights the Connolly surface with respect to a probe radius of 1.55 Å. (d) CO_2_ adsorption–desorption isotherms at 273 K. (e) Pore size distributions derived from CO_2_ sorption based on DFT calculations. (f) Methanol vapor sorption isotherms of polymer powders at 25 °C. Solid symbols in (d, f): adsorption; open symbols: desorption.

The target redox-active PIM polymer (PIM-TMTrip-MQ) was synthesized using a diamine monomer based on triptycene monobenzoquinone that contains tetramethyl groups to prohibit rotation around the C–N single bond (Figures 2a,b and S1 and S2). Additional experimental details, materials, and methods are provided in the Supporting Information. To compare PIM-TMTrip-MQ with structurally similar polymers, which contain imide and quinone functionalities but with a less microporous or nonporous structure, PI-Trip-MQ and PI-AQ were prepared (Figure 2a). PI-Trip-MQ is analogous to PIM-TMTrip-MQ but without tetramethyl groups, so that free rotation of imide bonds will lead to more efficient packing of polymer chains, isolating the internal molecular free volume elements generated by triptycene units from one another. Details of the synthesis of both triptycene monomers are provided in the Supporting Information. In contrast to the triptycene-based polyimides, the anthraquinone and diimide structural units in PI-AQ have a planar shape and are linked via imide linkages with unrestricted rotation freedom; hence, the formation of any free volume elements is reduced to a minimum. During the polymerization reaction, a highly viscous, homogeneous brown solution was obtained for PIM-TMTrip-MHQ, while yellow precipitate was immediately formed and remained insoluble throughout the reaction for PI-Trip-MHQ and PI-AQ. After precipitation and purification to remove oligomers, PIM-TMTrip-MHQ can be readily dissolved in polar aprotic solvents (such as NMP, DMF, and DMSO) owing to its rigid and contorted chain structure that reduces interchain cohesion and facilitates the absorption of solvent, enabling the fabrication of mechanically robust films by solution casting (Figure 1d). In contrast, PI-Trip-MHQ and PI-AQ are insoluble in common solvents, presumably due to the strong interchain π–π stacking interactions promoted by rearrangement and rotation of the more flexible polymer chains. Polymer structures were confirmed by FT-IR, solid-state ^13^C NMR, and TGA (Figures S3–S5). The incorporation of redox-active sites in both the structural unit and the linking group provides high theoretical capacity values of 189 mAh g^–1^ for PIM-TMTrip-MQ, 209 mAh g^–1^ for PI-Trip-MQ, and 248 mAh g^–1^ for PI-AQ based on a reversible four-electron redox reaction.

High uptake of nitrogen at low relative pressure at 77 K indicates freely accessible microporosity in PIM-TMTrip-MQ with a high apparent Brunauer–Emmett–Teller (BET) surface area value of 702 m^2^ g^–1^ (N_2_ adsorption isotherms and pore size distributions in Figure S6). In contrast, PI-Trip-MQ displayed negligible nitrogen uptake and an apparent BET surface area of only 6.4 m^2^ g^–1^. NLDFT pore size distribution derived from the nitrogen sorption isotherm confirms the absence of any micropores in PI-Trip-MQ that are accessible to nitrogen. Microporosity is consistent for PIM-TMTrip-MQ and PI-Trip-MQ with their hydroquinone precursors (labeled as MHQ, Figures S2 and S6). Interestingly, substantial carbon dioxide (CO_2_) adsorption at 273K (Figure 2d) was observed in the low-pressure range for PI-Trip-MQ with a value comparable to that for PIM-TMTrip-MQ, while its overall adsorption capacity is moderately lower. DFT pore size distributions calculated from these data gave a high concentration of both small (<0.7 nm) and large (0.7–1.0 nm) micropores in PIM-TMTrip-MQ, but only small ultramicropores (<0.7 nm) in PI-Trip-MQ (Figure 2e). The molecular simulation further verifies the presence of micropores in both PIM-TMTrip-MQ and PI-Trip-MQ, with the latter being predominantly ultramicroporous and less accessible to larger molecules (Figure 2c and simulation data in Figures S7–S9). Importantly, pore size distributions derived from molecular models agree well with those from the DFT analysis of CO_2_ sorption (Figures 2e and S8). It can be concluded that a continuous network of interconnected free volume elements (both small and large) is formed in PIM-TMTrip-MQ owing to its chain rigidity and contortion. In contrast, the absence of any restriction for C–N bond rotation in PI-Trip-MQ leads to more efficient packing of the polymer chains, generating predominantly ultramicropores that are only accessible to smaller carbon dioxide molecules at a higher temperature (273 K) but not to larger nitrogen molecules. In addition, the lowest carbon dioxide adsorption in PI-AQ that is composed of planar structural units further verifies triptycene monobenzoquinone as a porosity-generating structural unit in PIM-TMTrip-MQ and PI-Trip-MQ (Figure 2d–e).

Percolation of electrolyte solvents within redox-active materials is crucial to the formation of ion-transport channels for fast diffusion of metal ions with a short response time during battery charging and discharging. Methanol was used to probe the accessibility of micropores to organic solvents and assess the corresponding solvent uptake capacity (Figure 2f). Methanol uptake correlates well with the microporosity determined from CO_2_ sorption and molecular simulation, with the most microporous PIM-TMTrip-MQ showing the highest sorption of 36.5 wt % and the nonporous PI-AQ showing the lowest of 19.0 wt % at 273 K and 116 mbar. In addition to the siphoning effect driven by the capillary forces from micropores, solvent percolation in these polyimides of varied microporosity is also influenced by polymer swelling.

### 2.2 Electrochemical Properties

**Figure 3:**
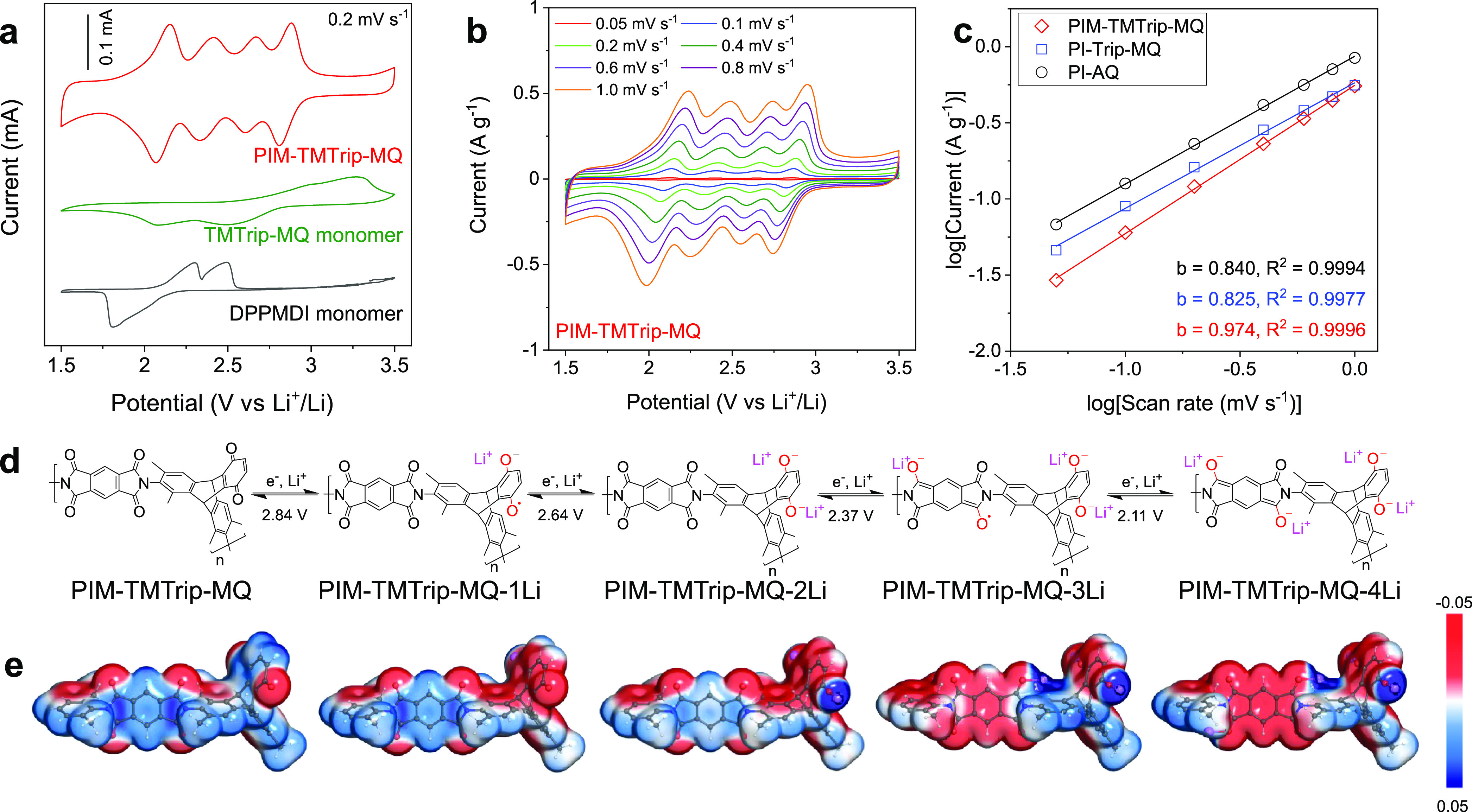
Electrochemical properties. (a) CV of PIM-TMTrip-MQ and model compounds TMTrip-MQ and DPPMDI. The electrolyte is a 1 M LiTFSI in DOL/DME (2:1 by vol.). (b) CV of PIM-TMTrip-MQ at varied scanning rates. (c) Log–log plot of peak current vs scanning rate for the anodic peak of the highest potential (∼2.9 V vs Li^+^/Li) in CV measurements in Figure S10. (d) Proposed redox mechanism of PIM-TMTrip-MQ. (e) Molecular electrostatic potential (MESP) of the PIM-TMTrip-MQ-nLi repeat units (*n* = 0–4).

Conventional coin cells with lithium foil anodes were prepared with cathodes fabricated from slurries of each polymer with carbon black and PVDF binder in anhydrous NMP. Solid-state cyclic voltammetry (CV) measurements showed that PIM-TMTrip-MQ exhibited four pairs of well-resolved redox peaks with *E*_1/2_ values of 2.11, 2.37, 2.64, and 2.84 V vs Li/Li^+^, respectively (Figure 3a,b). These redox peaks correspond to the transfer of two electrons in benzoquinone units and another two electrons in diimide units. In principle, the four carbonyls in diimide could accept four electrons to generate reduced tetra-anions as suggested by the calculated molecular electrostatic potential (MESP, Figures 3e and S13, Table S1). However, in previous work on other diimide polymers, the utilization of all four carbonyls has been shown to result in structural damage to the polymer,^38,39^ which does not appear to be occurring within the voltage range in this study. Similar CV curves as for PIM-TMTrip-MQ were observed in PI-Trip-MQ, but with broader peak widths and larger cathodic peak-to-anodic peak separation (Δ*E*_p_, Figure S10). Even larger peak separation was observed in PI-AQ, which showed broad and overlapping peaks at a scanning rate greater than 0.2 mV s^–1^. The difference in Δ*E*_p_ suggests larger polarization and lower utilization of carbonyl groups in the less porous PI-Trip-MQ and PI-AQ than in PIM-TMTrip-MQ, while the stronger intramolecular electronic interactions for the less porous polymers may also explain the broad and poorly resolved peaks.

Model compounds 2,6-diphenyl-pyromellitic diimide (DPPMDI) and tetramethyl-triptycene monobenzoquinone (TMTrip-MQ) were prepared to confirm the assignment of CV peaks of PIM-Trip-MQ (synthetic details in the SI). Solid-state CV measurements of the model compounds suggest that the imide units were reduced at less positive potentials of 2.06 and 2.25 V vs Li/Li^+^ in comparison to the more positive potentials required to reduce triptycene monobenzoquinone (2.58 and 2.83 V vs Li/Li^+^, Figure 3a). Therefore, we proposed a redox mechanism involving the successive, reversible conversions between the pristine PIM-TMTrip-MQ (fully oxidized state) and its tetra-anion derivative (fully reduced state) via the formation of quinone radical anions, quinone dianions, diimide radial anions, and diimide dianions (Figure 3d). This reaction mechanism is supported by DFT calculations on a cluster model of the hydrogen-capped PIM-TMTrip-MQ repeat unit (Figure S13 and Table S1). The molecular electrostatic potential (MESP) for the bare hydrogen-capped PIM-TMTrip-MQ repeat unit was first calculated to predict the possible Li binding sites. The surface minima revealed the carbonyl oxygens to be the most preferable sites for lithiation, where areas of high electron density were most likely to attract a Li^+^ ion. Based on the possible Li binding sites, a conformational search was then performed for the hydrogen-capped PIM-TMTrip-MQ repeat unit with different numbers of Li (*n* = 1–4, Figure S11). The lithiation pathway was predicted based on the minimum energy principle, and MESP plots were calculated for each preferred intermediate (Figure 3e). Predicted redox potentials in the solution phase fitted well with the values measured experimentally (Table S1), while the 5th and 6th lithiation of the PIM-TMTrip-MQ repeat unit, theoretically forming the diimide tetra-anions, showed considerably less positive predicted reduction potential, providing further evidence to support the proposed four-electron redox mechanism.

To understand the influence of polymer microporosity on Li^+^ ion diffusion and charge transfer in the composite electrodes, the log–log plots of the scanning rate versus the peak current density based on the CV measurements (Figure 3b) were analyzed. These parameters follow the power law in eq 1
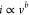
1Where *b* refers to the slope of the linear fit of the log–log plot. A *b* value approaching unity suggests a completely surface-controlled behavior during the electrochemical oxidation and reduction processes, while a value of 0.5 indicates a purely diffusion-controlled Randles–Ševčik behavior.^40^ PIM-TMTrip-MQ displayed a close-to-unity value of 0.974 (Figure 3c), much higher than those for PI-Trip-MQ (0.825) and PI-AQ (0.84). The high *b* value suggests that the rate limitation arising from diffusion constraints is effectively overcome in PIM-TMTrip-MQ with highly interconnected micropores, confirming the hypothesis that microporosity promotes ion diffusion in the redox-active polymers. This observation is further confirmed in acetonitrile electrolyte solutions with LiClO_4_, NaClO_4_, or TBAClO_4_ as the salt (Figure S11). Fast Li^+^ ion diffusion in the most microporous PIM is also supported by its high ionic conductivity as measured with a solution-cast film using electrochemical impedance spectroscopy (EIS) with values ranging from 1.7 to 2.7 mS cm^–1^ over the temperature range of 30–80 °C (Figure S14). The defect-free structure of the films used for the conductivity test was confirmed by single gas permeation measurements (Table S2). However, we were not able to process PI-Trip-MQ and PI-AQ into dense films due to their poor solubility in organic solvents, precluding the direct comparison of their ionic conductivity.

### 2.3 Stable Cycling Performance Enabled by Solution-Processed Electrode

**Figure 4:**
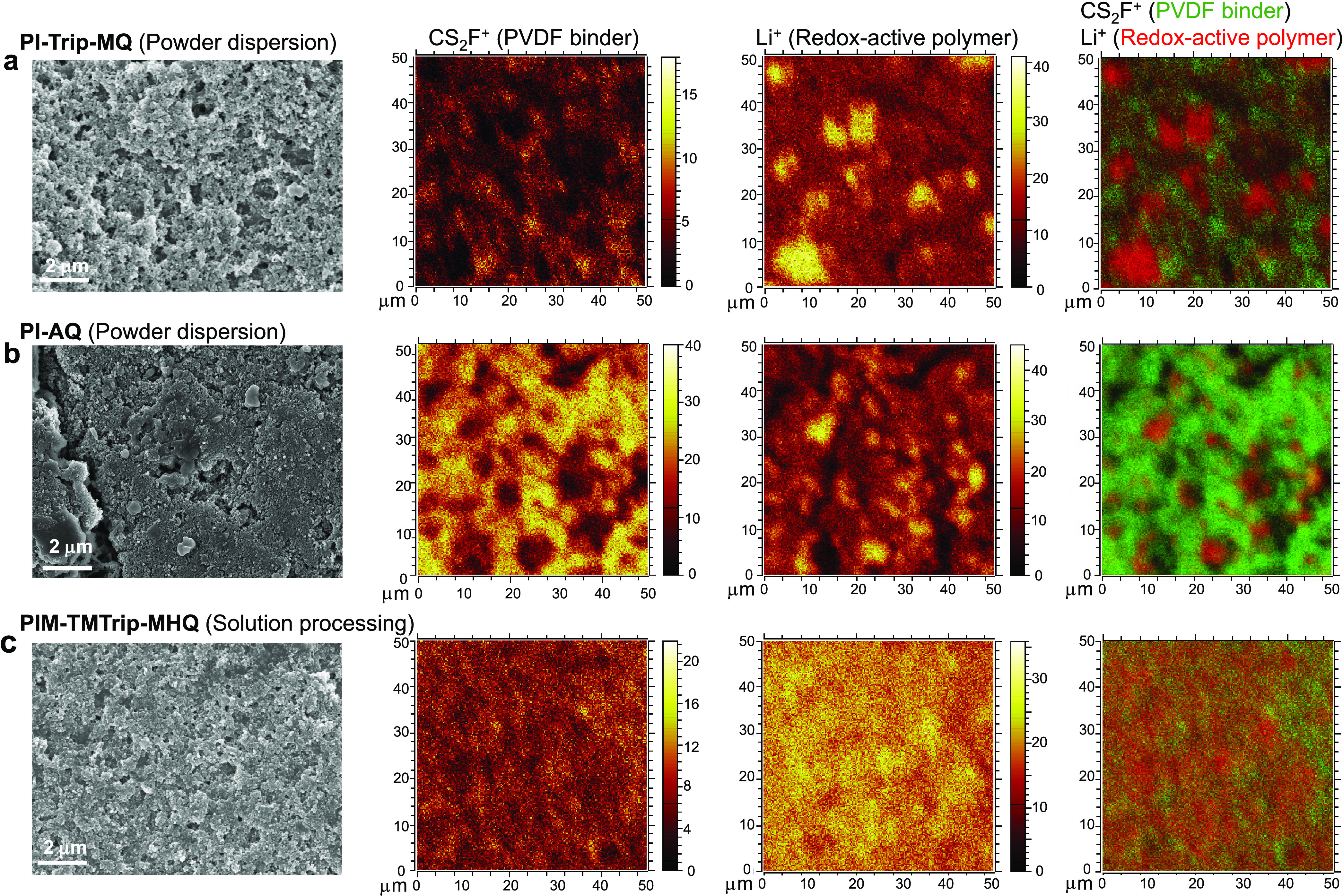
Characterization of electrode microstructure. SEM and ToF-SIMS images for (a) PI-Trip-MQ and (b) PI-AQ electrodes that were prepared by powder dispersion and (c) PIM-TMTrip-MHQ electrodes prepared by solution processing. The electrodes were disassembled from batteries at an SOC of 0 (fully discharged) and then thoroughly washed with DOL/DME to remove absorbed lithium salts, followed by drying. First column: SEM images. Second and third columns: secondary ion images showing the distribution of PVDF binder (Cs_2_F^+^) and polymer (Li^+^), respectively. Fourth column: combined secondary ion images showing the spatial distribution of PVDF (green) and redox-active polymer (red).

As most redox-active organic materials are insulating, conductive additives are required to form a composite with the active materials. It is crucial to control the microstructure of the composites to achieve efficient ion transport and electron transfer and to avoid heterogeneous current density distribution that is linked to reduced accessible capacity and local overcharge^41−43^ (Figure 1e,f). We initially applied the conventional powder dispersion method to fabricate insoluble PI-Trip-MQ and PI-AQ electrode composites by manually grinding each polymer powder, carbon black, and the PVDF binder with NMP added to form a slurry, which was then cast onto alumina foil and dried under vacuum. SEM images showed discrete large polymer flakes or particles as well as carbon aggregates (Figures 4a,b and S15). Time-of-flight secondary ion mass spectrometry (ToF-SIMS) further revealed the heterogeneous spatial distribution of different components in the electrode composites, as visualized by the signals of lithium (Li^+^) and fluorine (Cs_2_F^+^) that correspond to redox-active polymers and PVDF binder, respectively. The spatial distributions of redox-active polymers and PVDF were complementary to each other, exhibiting scattered large polymer particles (1–10 μm in diameter) embedded in the PVDF matrix (Figure 4a,b). Although the distribution of carbon black aggregates cannot be measured due to its lack of characteristic fragments, we envision that PVDF binder forms a coating layer on carbon black particles, and thus, the spatial distribution of carbon blacks would be consistent with that of the PVDF binder.

The solubility of PIM-TMTrip-MHQ in NMP further motivated us to modify the fabrication method to achieve a more homogeneous microstructure in the composite electrode. The modified method included dissolving the polymer and PVDF binder in NMP to form a homogeneous solution within which carbon black was then dispersed, followed by the identical casting and drying procedures as in the powder dispersion method. No large particles or flakes could be identified from SEM images (Figures 4c and S16), while the apparent size of the carbon black particles was noticeably larger than that in a control sample fabricated using only carbon blacks and PVDF binder (Figure S16c). ToF-SIMS showed homogeneous spatial distribution of the redox-active polymer and PVDF binder across the electrode composite (Figure 4c), confirming the well-defined microstructure enabled by solution processing. The uniform coating of PIM-TMTrip-MHQ onto the carbon additives achieves intimate contact and seamless interfaces between different components, allowing the formation of an ionic and electronic percolation network, which is the key to ensuring the homogeneity of current distribution within the electrodes.

**Figure 5:**
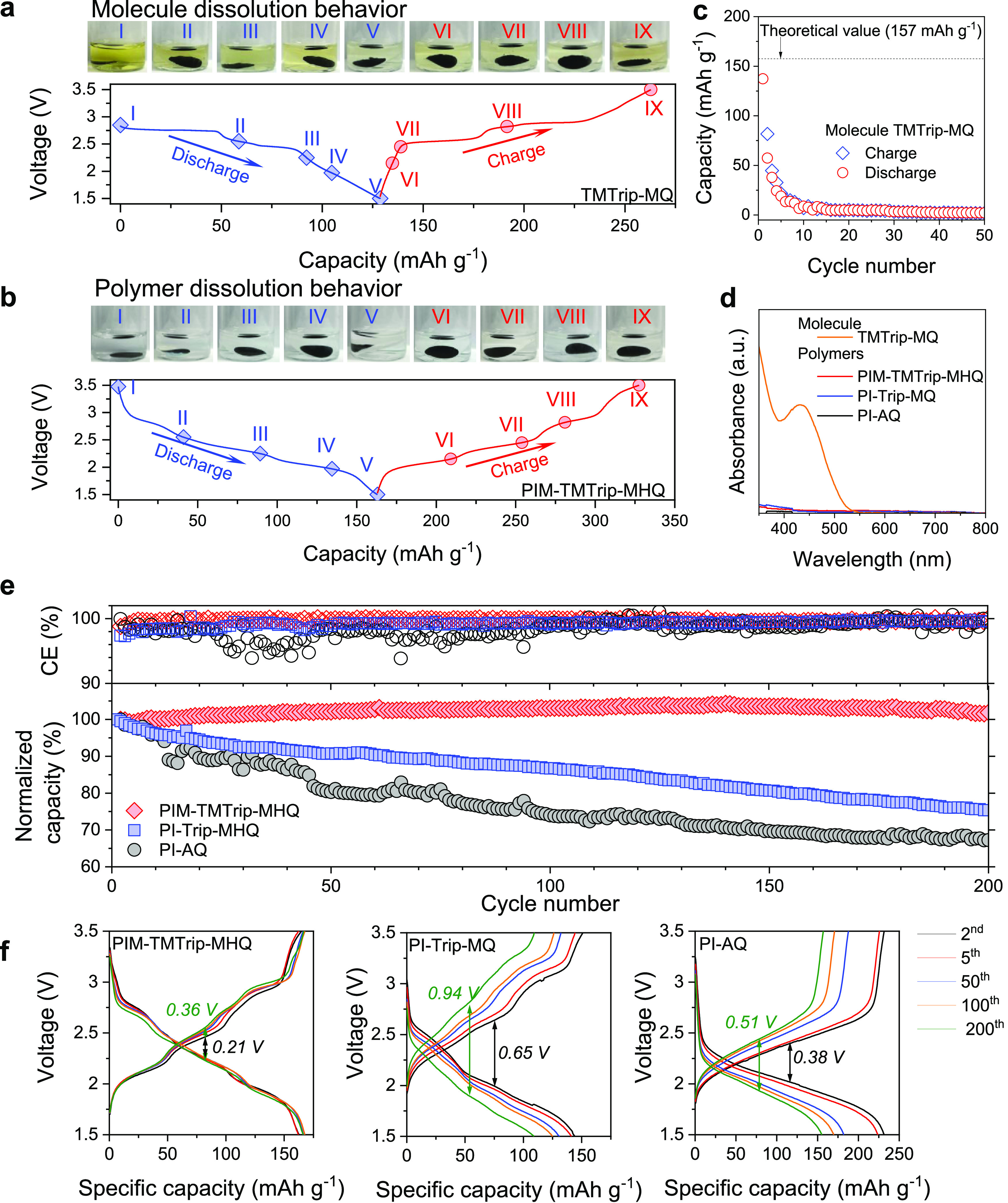
Cycling performance of lithium-ion batteries. (a, b) Solubility tests of electrodes at varied states of charge for a molecular compound TMTrip-MQ (a) and PIM-TMTrip-MHQ polymer (b). (c) Cycling performance for a TMTrip-MQ-based LIB, showing rapid capacity decay due to molecule dissolution and shuttling. (d) UV–vis spectra of the washing solution for electrodes disassembled from LIB cells after discharge. (e) LIB cycling performance for redox-active PIMs. (f) Charging–discharging profiles at different cycling numbers.

After microstructure optimization of the polymer–carbon composites, their performance as the cathode in lithium-ion batteries was evaluated. To study the dissolution and shuttling of redox-active materials as the capacity decay mechanism, we performed *ex situ* solubility tests of electrodes disassembled from cells at different states of charge (Figure 5a,b). The small molecule, TMTrip-MQ, was found highly soluble in electrolytes regardless of the state of charge, leading to complete loss of capacity within 10 cycles of charging and discharging (Figure 5c). In contrast, all polymers remained insoluble in battery electrolyte solvents, as confirmed by UV–vis (Figure 5d). The cell based on a solution-processed PIM-TMTrip-MHQ cathode demonstrated highly stable cycling performance with a Coulombic efficiency (CE) of 99.8% and no apparent capacity decay over 200 charging–discharging cycles at 1C (Figures 5e and S19 and S20). The cell polarization remained low throughout the cycling test (Figure 5f). In contrast, otherwise-identical cells based on PI-Trip-MQ and PI-AQ cathodes that were prepared by the powder dispersion method exhibited much larger cell polarization and a steady capacity decay with a CE of 98.3 and 99.2% and capacity retention of 75 and 67%, respectively, after 200 charging–discharging cycles at 1C (Figure 5e); such performance decay is commonly observed for polymer-based electrodes fabricated by the powder dispersion method. One potential decay mechanism for PI-Trip-MQ and PI-AQ is the irreversible side reactions and structural changes of active materials induced by local overcharge,^41−43^ while the well-defined microstructure of the solution-processed PIM-TMTrip-MHQ electrode appears to ensure homogeneous current distribution that prevents capacity loss, as evidenced by the uniform spatial distribution of Li^+^ ions in the electrode after discharge. Furthermore, the PI-Trip-MQ and PI-AQ electrodes may undergo delamination of the discrete polymer particles with carbon additives and the current collector due to volume changes of polymer particles during charging and discharging, while the solution-processed electrodes possess a certain level of resistance toward the delamination between polymer coating layer with carbon cores.

**Figure 6:**
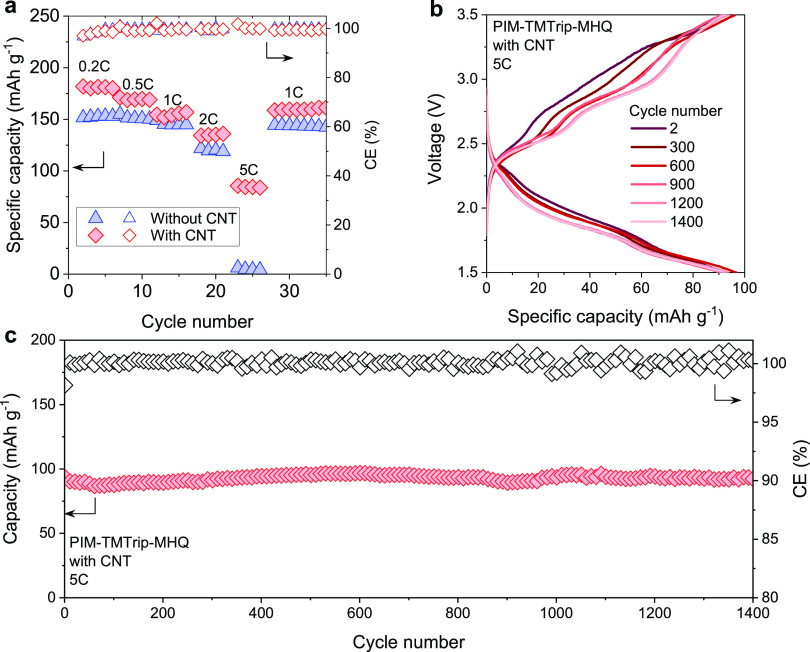
Rating performance improvement enabled by carbon nanotube (CNT) additives. (a) Rating performance of PIM-TMTrip-MHQ electrodes with or without CNT additives. (b) Charging–discharging profiles at different cycling numbers and (c) LIB cycling performance for PIM-TMTrip-MHQ electrode with CNT additives.

In general, redox-active organic materials have relatively poor electronic conductivity aside from doped conductive polymers^44^ and π-conjugated redox polymers.^45^ Redox-active PIMs are not an exception in this regard, showing through-plane electronic conductivity values of 2.9 × 10^–13^, 2.3 × 10^–12^, and 2.5 × 10^–14^ S cm^–1^ for PIM-TMTrip-MQ, PI-Trip-MQ, and PI-AQ, respectively. These values are within typical ranges for carbonyl, imine, and radical-based materials that do not contain delocalized π-electron systems as conductive moieties.^1^ As a result, redox-active PIMs showed limited rating performance, with the discharge capacity dropping to negligible at 5C (Figures 6a and S20), while removing carbon additives from the composite electrode led to the “shut-down” of redox activity (Figure S12). In contrast, some carbonyl polymers are demonstrated with high capacity at high charge and discharge rates, such as poly(1,4-anthraquinone)^5^ which delivers 69% of their low-rate capacity (181 mAh g^–1^) at 20C as well as a lithium salt polymer of dihydroxyanthraquinone^46^ that displays the capacity of 236 mAh g^–1^ at 10C. The high rating performance of these polymers is based on their high electronic conductivity, which is ensured by the π-conjugated structure. To improve electron transfer within the composite electrode, we further utilized carbon nanotubes (CNT) as conductive additives to construct a more conducting network. The discharge capacity of PIM-TMTip-MHQ/CNT composite electrode was improved to the range of 80–90 mAh g^–1^ at 5C from none for the electrode without CNT (Figure 6b), but the improvement is highly dependent on mass loading (Figure S21). Importantly, the solution-processed electrode exhibited a stable cycling performance over 1400 cycles of charge and discharge, further confirming the important role of solution processing in achieving high-capacity retention (Figure 6c). To track the evolution of the electrochemical properties of PIM-TMTrip-MHQ electrode during cycling, we performed incremental capacity analysis (Figure S22) based on the differentiation (d*Q*/d*V*) of the charging–discharging profiles, a method widely used as a diagnostic tool to investigate battery state of health. The 2nd cycle of charging showed four well-resolved peaks in the incremental capacity curve at 2.47, 2.81, 3.07, 3.31 V, respectively, in good agreement with CV measurements. However, the two peaks at lower potentials (i.e., 2.47 and 2.81 V) underwent a continuing increase in peak area during cycling, accompanied by a shift of redox potential toward higher values (i.e., the cutoff voltage of 3.5 V) and a decrease in peak area for the other two peaks. The mechanism for the change of potential plateaus during cycling merits further study.

### 2.4 Discussion on Rating Performance and Charge Capacity

Reaction kinetics in electrode materials is controlled by ionic transport, electron transfer as well as ionic–electronic coupling. The influence of ionic transport has been extensively studied for porous network polymers, particularly covalent organic frameworks (COFs). As the electron transfer in COFs is usually efficient, superior rating performance can be achieved through the enhancement of ionic transport. For conventional nonporous conjugated polymers, the high degree of π-conjugation ensures efficient intrachain electron movement through delocalized π-orbitals and interchain electron movement where there is sufficient π–π overlap between polymer chain segments. Ion transport in these nonporous polymers mainly relies on electrolyte swelling and could be sluggish for some polymer chemistries. For redox-active PIMs reported in this work, ionic transport is effectively enhanced by their intrinsic micropores; however, their nonconjugated structure leads to inferior electron transfer. Consequently, the apparent redox kinetics and rate performance of redox-PIMs are found poorer than some previously reported COFs and conventional conjugated polymers. Nevertheless, strategies to enhance ionic transport and improve battery lifetime, even when electron transfer is limited, broaden the scope of the potential materials that can be used in energy storage, and may inspire the development of better materials that show optimal performance in all aspects of the evaluating matrix. We envision that optimal performance would be achieved if (1) redox-active PIMs are employed in systems where ion uptake and transport are more critical (e.g., multivalent metal-ion batteries^47^), (2) greater electronic conjugation could be introduced to these microporous polymers, or (3) the concept of PIMs is extended to other redox chemistries where electronic conjugation is not a prerequisite for high performance, such as organic radical polymers.^48,49^

Although all structural units employed in our material design are redox active, there are still inactive segments present in the structural units of redox-active PIMs (e.g., two benzo groups of the triptycene benzoquinone component). Further enhancement of specific capacity could be achieved based on recent progress in making multielectron redox quinone materials including pillarquinone macrocycles^50^ and polymers containing pyrene-4,5,9,10-tetraone^51^ and anthratetronyl sulfide.^46^

## 3 Conclusions

The design and synthesis of redox-active PIMs with accessible microporosity and high specific capacity involves, first, the use of a triptycene benzoquinone structural unit to achieve a high density of redox-active sites while maintaining the highly rigid and contorted structure, and second, the introduction of tetramethyl groups to provide a sufficient steric hindrance to restrict rotation about the C–N imide single bond. Importantly, these redox-active PIMs can be readily solution-processed into thin films and electrodes with a well-defined microstructure, enabling enhanced Li^+^ diffusion and stable cycling performance in lithium-ion batteries. The strategy of designing redox-active PIMs with combined properties of intrinsic microporosity, reversible redox activity, and solution processability may inspire the development of redox-active polymers with applications in a variety of electrochemical devices for energy storage, electrochemical sensors, supercapacitors, and photonic devices.

## Supporting Information Available

Additional experimental details, materials, and methods, including the synthesis of all compounds; simulation methods; FT-IR; TGA; NMR; N_2_ adsorption; simulation results; CV; ion conductivity; gas permeability; SEM images; battery performance data; and analyses (PDF)
