# Supplementary material for: Solution-Processable Redox-Active Polymers of Intrinsic Microporosity for Electrochemical Energy Storage
Source: J Am Chem Soc. 2022 Sep 8;144(37):17198–208. doi: 10.1021/jacs.2c07575 (PMC9501925; doi:10.1021/jacs.2c07575)
Supplement: Supplementary file 1 — ja2c07575_si_001.pdf [file ja2c07575_si_001.pdf]

Solution-processable redox-active polymers of intrinsic microporosity for electrochemical energy storage

Anqi Wang[1†], Rui Tan[1†], Charlotte Breakwell[2], Xiaochu Wei[1], Zhiyu Fan[1], Chunchun Ye[3], Richard Malpass-Evans[3], Tao Liu[4], Martijn A. Zwijnenburg[5], Kim E. Jelfs[2], Neil B. McKeown[3*], Jun Chen[6], Qilei Song[1*]

[1]Department of Chemical Engineering, Imperial College London, London SW7 2AZ, U.K.

[2]Department of Chemistry, Molecular Sciences Research Hub, Imperial College London, London W12 0BZ, U.K.

[3]EaStChem School of Chemistry, University of Edinburgh, Edinburgh, EH9 3FJ, U.K.

[4]Shanghai Key Laboratory of Chemical Assessment and Sustainability, Department of Chemistry, Tongji University, Shanghai, China.

[5]Department of Chemistry, University College London, London, WC1H 0AJ, U.K.

[6]Key Laboratory of Advanced Energy Materials Chemistry (Ministry of Education), Renewable Energy Conversion and Storage Center (RECAST), College of Chemistry, Nankai University, Tianjin, China.

[†]These authors contributed equally.

Email: Neil.McKeown@ed.ac.uk, q.song@imperial.ac.uk

Contents

1. General methods and equipment....................2

2. Synthesis of monomers, polymers and model compounds....................3

3. Electrochemical measurements....................8

4. Computational Methods....................10

1. General methods and equipment

Commercially available reagents were used without further purification. Multi-walled carbon nanotubes (outer diameter: 5-15 nm; length 10-30 μm) were purchased from Jiangsu XFNANO Materials Tech. Co.,Ltd. All reactions using air/moisture sensitive reagents were performed in oven-dried apparatus under a nitrogen atmosphere.

Scanning electron microscopy (SEM) was performed using a LEO Gemini 1525 FEGSEM for fresh electrodes or a Zeiss Gemini Sigma300 for post-cycling electrodes. Samples were coated with a thin layer of chromium or gold. ***Infrared spectroscopy*** was performed on a Perkin-Elmer Spectrum 100 FTIR spectrometer with polymer samples mounted on a zinc–selenium/diamond plate. ***Thermal analyses*** were performed using a PerkinElmer TGA 8000 thermogravimetric analyser. Samples were heated from room temperature to 1000 °C under flowing nitrogen at a heating rate of 10 °C min^{-1}. ***High-resolution mass spectrum*** (HRMS) was obtained in electron impact ionization (EI) mode on a Thermo Scientific Mat 900 XP double focus sector mass spectrometer. ***ToF-SIMS*** analysis was performed using an ION-TOF ToF-SIMS V instrument. Lithium-ion battery cells were subjected to activation and one cycle of charging-discharging at 1C and then disassembled in a glove box to obtain electrode samples in their lithiated (discharged) state. To remove absorbed LiTFSI salts, the electrode samples were soaked in 3 mL DOL solvent three times with each time taking at least 4 hours, followed by drying under vacuum. ***Low-pressure gas physisorption*** was performed using a Micromeritics 3Flex surface characterization analyser. Each sample was degassed at 110 °C under vacuum for 12 h, and then loaded into the apparatus and in situ degassed at 110 °C for another 3 h. Nitrogen adsorption isotherms were measured at 77 K, and carbon dioxide adsorption isotherms were measured at 273 K. ***Dynamic methanal vapour sorption*** was performed using an IGA-001 gravimetric sorption analyzer (Hidden isochema) at 25 °C. Polymer samples (20–30 mg) were *in situ* dried in vacuo at 110 °C for at least 12 h until the mass became constant. ***UV–Vis spectroscopy*** was measured using a UV–Vis spectrometer (UV-1800, Shimadzu) with a wavelength range of 200–800 nm at an interval of 0.5 nm. High-powered decoupling magic angle spinning ^{13}C solid-state NMR spectra were collected using a Bruker Avance III 600 MHz instrument using an adamantane reference. A spinning rate of ~15,000 Hz was used with powder samples packed into a 3.2 mm zirconium rotor. Spectra were typically compiled from ~4,000 scans with a 6 s recycle delay. Electrode samples for ***solubility tests*** (Fig 5 **a**-**b**) were prepared by disassembling the electrodes and Celgard separator from battery cells at a given potential and soaking in 2 mL electrolyte solution overnight before UV-Vis measurements.

Single gas permeation was measured at a feed pressure of 4 bar and 22°C, using a homemade constant-volume-variable-pressure apparatus described elsewhere[1]. The Membrane sample was soaked in methanol overnight and then annealed at 110°C under vacuum for 12 h prior to measurements.

2. Synthesis of monomers, polymers and model compounds

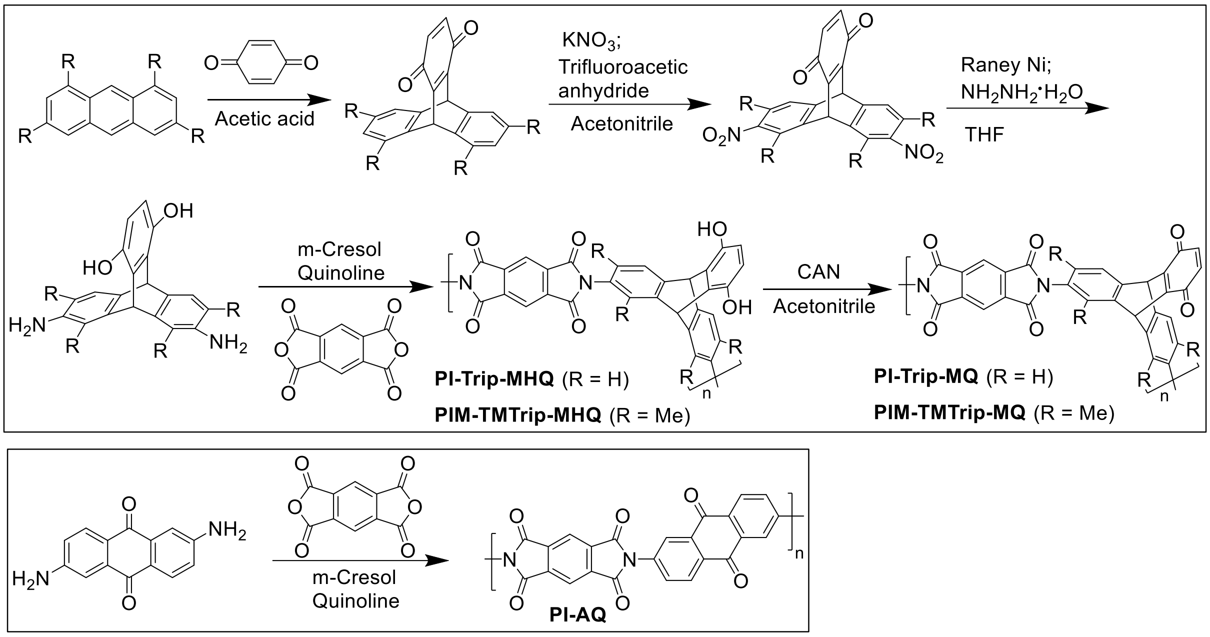

Fig. S1. Synthesis of redox-active PIMs.

<u>1,3,6,8-tetramethylanthracene</u>

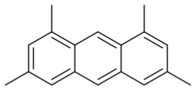

Anhydrous dichloromethane (40 mL, 0.63 mol) was added dropwise to a suspension of aluminium trichloride (60 g, 0.45 mol) in anhydrous *m*-Xylene (200 mL, 1.62 mol) at 0 ºC over 1 h. The mixture was heated at 60 ºC for 3 h and then at 80 ºC for 5h, cooled and poured into crushed ice. The organic layer was extracted with diethyl ether and the solvent removed under vacuum at 80 ºC to afford a green solid. The crude product was purified by flash chromatography over silica gel with petroleum ether (40-60 ºC) to yield 1,3,6,8-tetramethylanthracene (28.0 g, 0.119 mol, 19%, Lit 23%[2]) as colourless crystals. ^{1}H NMR (500 MHz, $CDCl_3$): δ (ppm) 8.52 (s, 1H), 8.20 (s, 1H), 7.60 (s, 2H), 7.15 (s, 2H), 2.81 (s, 6H), 2.52 (s, 6H). ^{13}C NMR (125 MHz, $CDCl_3$): δ (ppm) 134.6, 134.4, 132.3, 129.4, 128.3, 125.4, 124.7, 118.7, 22.0, 19.8. HRMS (EI, m/z): [M^+] calculated: 234.14069, found: 234.14193

(9s, 10s)-9,10-dihydro-9,10-[1,2]benzenoanthracene-13,16-dione (Trip-MQ)

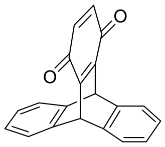

Acetic acid glacial (250 mL) was added to anthracene (10.07 g, 56.5 mmol) and 1,4-benzoquinone (10.6g, 98.0 mmol). The mixture was heated to reflux for 3 h and then poured into water. The precipitate was filtered and washed with hot water. The crude product was purified by column chromatography over silica gel with DCM to give Trip-MQ (10.0 g, 35.2 mmol, 62%) as a yellow powder. ^{1}H NMR (500 MHz, $CDCl_3$): δ (ppm) 7.44-7.42 (m, 4H), 7.05-7.03 (m, 4H), 6.60 (s, 1H), 5.80 (s, 1H); ^{13}C NMR (125 MHz, $CDCl_3$): δ (ppm) 183.5, 151.9, 143.6, 135.4, 126.6, 124.4, 47.4. HRMS (EI, m/z): [M^+] calculated: 284.08178, found: 284.08250.

1,3,6,8-tetramethyl-9,10-dihydro-9,10-[1,2]benzenoanthracene-13,16-dione (TMTrip-MQ)

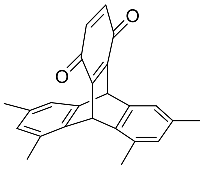

Acetic acid glacial (28 mL) was added to 1,3,6,8-tetramethylanthracene (1.03 g, 4.4 mmol) and 1,4-benzoquinone (4.54 g, 42 mmol). The mixture was heated to reflux for 3 h and then poured into water. The precipitate was collected by filtration and washed with hot water. The crude product was purified by flash chromatography over silica gel with dichloromethane/petroleum ether 40-60 ºC (1:1, v/v) to yield (1.06g, 3.1 mmol, 70%) as an orange powder. ^{1}H NMR (500 MHz, $CDCl_3$): δ (ppm) 7.09 (s, 2H), 6.68 (s, 2H), 6.57 (s, 2H), 6.18-5.64 (m, 2H), 2.46 (6H), 2.22 (2H). ^{13}C NMR (125 MHz, $CDCl_3$): δ (ppm) 183.9, 152.6, 144.3, 139.4, 135.5, 132.9, 127.7, 123.2, 48.0, 44.0, 39.9, 21.0, 18.6. HRMS (EI, m/z): [M^+] calculated: 340.14480, found: 340.13196.

(9R,10R)-2,6-((9s,10s)-2,7-)dinitro-9,10-dihydro-9,10-[1,2]benzenoanthracene-13,16-dione (DNTrip-MQ)

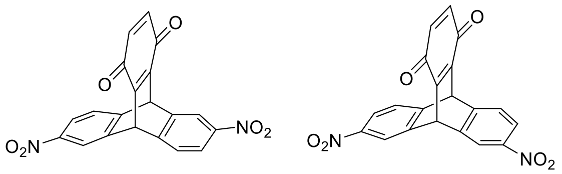

Trifluoroacetic anhydride (11 mL, 79 mmol) was added dropwise to a mixture of potassium nitrate (1.02 g, 10.1 mmol) and Trip-MQ (1.43g, 5.0 mmol) in acetonitrile (24 mL). The

mixture was stirred at room temperature for 24 h. The solvent was removed under vacuum, and the residue was stirred in water and then extracted with chloroform. The crude product was subjected to column chromatography over silica gel with dichloromethane/petroleum ether 40-60 °C (7:3, v/v) to afford DNTrip-MQ (1.20g, 3.2 mmol, 64%) as a yellow powder. ^{1}H NMR (500 MHz, $CDCl_3$): δ (ppm) 8.44/8.38 (d, 2H, J = 2.3 Hz), 8.03/8.01 (t, 2H, J = 1.9 Hz), 7.84 (d, 2H, J = 3.3 Hz)/7.82 (d, 2H, J = 3.3 Hz), 6.79 (s, 2H), 6.36(s, 2H)/6.40(s, 1H) and 6.32(s, 1H); ^{13}C NMR (125 MHz, $CDCl_3$): δ (ppm) 182.7, 182.6, 150.1, 149.7, 149.5, 148.9, 145.4, 145.0, 144.6, 135.6, 125.7, 125.6, 122.0, 119.7, 119.5, 46.1, 45.9, 45.7. HRMS (EI, m/z): [M^+] calculated: 374.05519, found: 374.05594.

1,3,6,8-tetramethyl-2,7-dinitro-9,10-dihydro-9,10-[1,2]benzenoanthracene-13,16-dione (TMDNTrip-MQ)

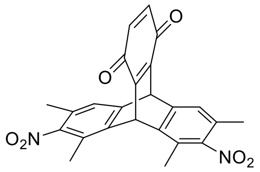

Trifluoroacetic anhydride (2.84 mL, 20.1 mmol) was added dropwise to a mixture of potassium nitrate (0.583 g, 5.76mmol) and TMTrip-MQ (0.98 g, 2.88 mmol) in acetonitrile (100 ml). The mixture was stirred at room temperature for 48 h. The solvent was removed under vacuum and residue was stirred in water then extracted with dichloromethane to afford a yellow solid. The crude product was purified by column chromatography over silica gel with dichloromethane/petroleum ether 40-60 °C (2:1, v/v) to yield TMDNTrip-MQ (0.80 g, 1.86 mmol, 65%) as a yellow powder. ^{1}H NMR (500 MHz, $CDCl_3$): δ (ppm) 7.22 (s, 2H), 6.67 (s, 2H), 6.27-5.74 (m, 2H), 2.43 (s, 6H), 2.22 (s, 6H). ^{13}C NMR (125 MHz, $CDCl_3$): δ (ppm) 182.9, 151.2, 151.0, 144.7, 140.8, 135.6, 127.6, 125.1, 47.6, 44.0, 40.2, 17.4, 13.9. HRMS (EI, m/z): [M^+] calculated: 430.11700, found: 430.12612.

(9R,10R)-6,15[-((9s,10s)-6,14-]diamino-9,10-dihydro-9,10-[1,2]benzenoanthracene-1,4-diol (DATrip-MHQ)

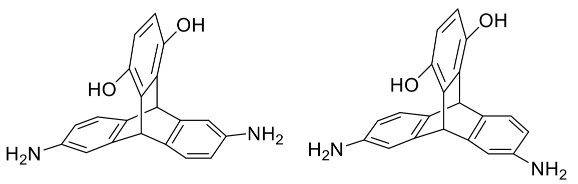

DNTrip-MQ (2.0 g, 5.3 mmol) was dissolved in de-oxygenated THF (100 mL) under N_2. Raney nickel (~50 mg) and hydrazine monohydrate (5.1 g, 102 mmol) was added dropwise to the solution. The mixture was heated to reflux for 24 h. The colourless mixture was cooled in ice

and filtered through Celite, and then the solvent was removed under vacuum. The residue was washed with water and chloroform, and dried under vacuum at 70 °C overnight to afford a white powder (1.55g, 4.9 mmol, 92%). ^{1}H NMR (500 MHz, $(CD_3)_2SO$): δ (ppm) 8.57 (s, 2H)/8.60 (s, 1H) and 8.54(s, 1H), 6.93/6.91 (d, 2H, J = 7.8 Hz), 6.62/6.59 (d, 2H, J = 2.1 Hz), 6.24/6.25 (s, 2H),6.09-6.06 (m, 2H), 5.34 (s, 2H)/5.35 (s, 1H) and 5.34 (s, 1H), 4.75 (s, 4H). HRMS (EI, m/z): [M^+] calculated: 316.12011, found: 316.11938**(9s,10s)-6,14-diamino-5,7,13,15-tetramethyl-9,10-dihydro-9,10-[1,2]benzenoanthracene-1,4-diol (DATMTrip-MHQ)**

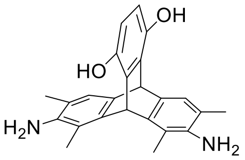

DNTMTrip-MQ (0.95 g, 2.2 mmol) was dissolved in de-oxygenated THF (150 mL) under N_2. Raney nickel (~50 mg) and hydrazine monohydrate (2.1 g, 42 mmol) was added dropwise to the solution. The mixture was heated to reflux for 24 h. The colourless mixture was cooled in ice and filtered through Celite, and then the solvent was removed under vacuum. The residue was washed with water and chloroform, and dried under vacuum at 70 °C overnight to afford a white powder (0.51g, 1.4 mmol, 64%). ^{1}H NMR (500 MHz, $(CD_3)_2SO$): δ (ppm) 8.51 (s, 1H), 8.44 (s, 1H), 6.75-5.29 (m, 4H), 6.21 (s, 2H), 4.19 (s, 4H), 2.24 (s, 6H), 1.96 (s, 6H). LRMS (EI, m/z): [M^+] calculated: 372.18261, found: 372.17897

2,6-diphenylpyrrolo[3,4-f]isoindole-1,3,5,7(2H,6H)-tetraone (DPPMDI)

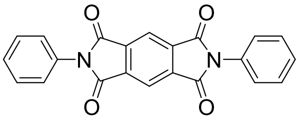

Aniline (3.53 g, 37.9 mmol) and PMDA (2.18 g, 10.0 mmol) were stirred in dry *m*-cresol (40 mL) under N_2 for 15 min before being heated to 80 °C. After the addition of toluene and 0.2 mL *iso*-quinoline, the reaction temperature was gradually raised to 200 °C in 1 h and then kept at 200 °C for 5 h. Water formed from the cyclo-imidization reaction was removed by forming an azeotrope with toluene aided by the Dean-Stark apparatus. After cooling to room temperature, the precipitate was collected by filtration, washed with hot DMF, methanol and acetone, and then dried at 110 °C for 24 h to afford DPPMDI (3.43 g, 9.31 mmol, 93%) as yellow crystals. The chemical structure of the product was not characterized due to its poor solubility in common solvents tested, which has been observed previously[3].

General procedure for the synthesis of polyimides

Diamine monomer and PMDA were stirred in dry *m*-cresol under N_2 at room temperature for 15 min before being heated to 80 °C. Toluene and 1 mL *iso*-quinoline were added to the reaction mixture and reaction temperature was gradually raised to 200 °C in 1 h. The reaction mixture was kept at 200 ºC for 4-5 h. Water formed from the cyclo-imidization reaction was removed by forming an azeotrope with toluene aided by the Dean-Stark apparatus. After cooling to room temperature, the mixture was added dropwise to methanol and the precipitate collected by filtration. After purification, the product was dried under vacuum at 110 ºC for 24 h to afford the product.

PIM-TMTrip-MHQ

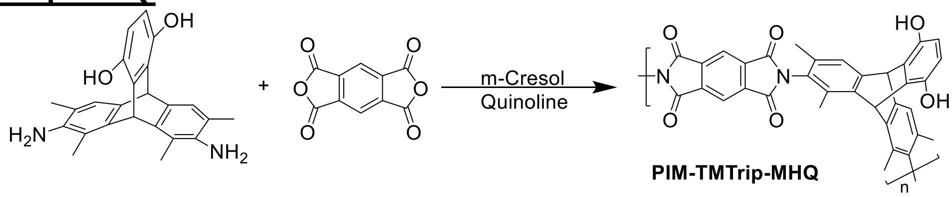

General procedure was followed using DATMTrip-MHQ (3.16 g, 8.48 mmol), PMDA (1.85 g, 8.48 mmol), 30 mL m-cresol, 0.2 mL iso-quinoline, and 10 mL toluene. Monomers were not fully dissolved during the initial reaction period until the temperature exceeded 80 ºC. The mixture was heated at 200 ºC for 4 h and turned into a highly viscous homogeneous brown solution. After cooling to room temperature, the solution was poured into methanol, and the precipitate was collected by filtration and purified by washing with hot methanol and acetone to afford PIM-TMTrip-MHQ (4.59 g, 8.22 mmol, 97%) as a light brown powder.

PI-Trip-MHQ

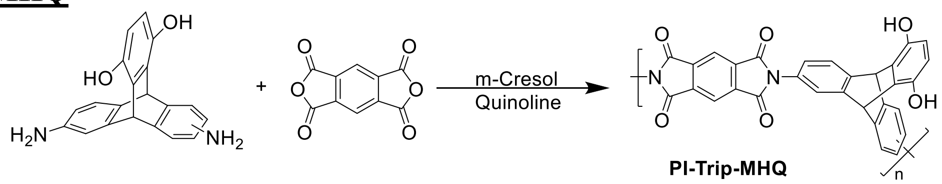

General procedure was followed using DATrip-MHQ (1.217 g, 3.85 mmol), PMDA (0.834 g, 3.83 mmol), 14 mL m-cresol, 0.2 mL iso-quinoline, and 10 mL toluene as well as purification by washing with hot DMF, methanol and acetone to afford PI-Trip-MHQ (1.82 g, 3.65 mmol, 95%) as a light brown powder.

PI-AQ

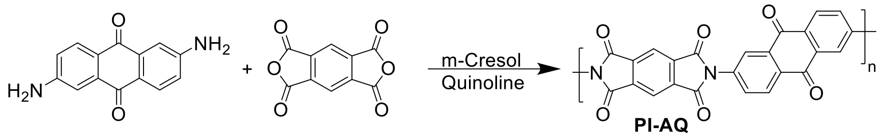

General procedure was followed using 2,6-diaminoanthraquinone (4.77 g, 20.0 mmol), PMDA (4.36 g, 20.0 mmol), 14 mL m-cresol, 0.2 mL iso-quinoline, and 10 mL toluene. The reaction mixture was heated at 200 °C for 5 h. After cooling to room temperature, the mixture was directly filtered to afford a dark brown powder and nearly colourless filter liquor. The crude product was purified by repeated stirring-filtration procedure in boiling DMF until the washing liquid changed from dark brown to colourless, followed by washing with methanol and acetone to yield PI-AQ (7.92 g, 18.8 mmol, 94%) as a red powder.

PIM-TMTrip-MQ/PI-Trip-MQ

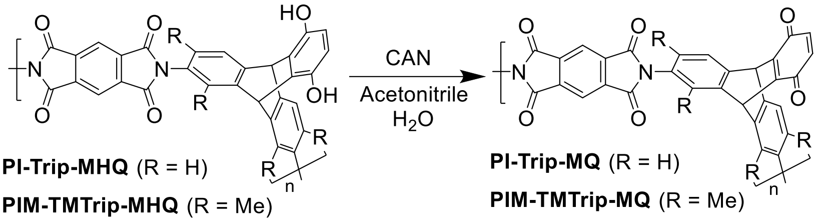

Ceric ammonium nitrate (CAN, 6.0 g, 11 mmol) was added to a suspension of PIM-TMTrip-MHQ (0.5 g, 0.91 mmol) or PI-Trip-MHQ (0.50 g, 1.0 mmol) in acetonitrile (100 mL) and water (10 mL). The mixture was stirred at room temperature for 24 h, and then filtered and washed with water to afford a yellow powder in quantitative yield.

3. Electrochemical measurements

Ionic conductivity of the solution-cast PIM-Trip-MHQ film was measured by electrochemical impedance spectroscopy (EIS) using the potentiostat mode with a perturbation of 10 mV and a frequency range of 0.5 MHz–10 Hz. The membrane sample was soaked in 1 M LiTFSI in DOL/DME (2:1 by Vol.) for 24 h prior to measurements, and then sandwiched between two stainless steel electrodes and sealed in coin cells (Type 2032). The ionic conductivity was calculated according to the following equation: $\sigma = L/(R_m \times A)$, where R_m is ionic resistance, L is membrane thickness and A is the active membrane area (2.00 cm^2). R_m equals the intercept of the Nyquist plots with x-axis. Membrane thickness was measured by a micrometer.

Electronic conductivity of the polymers was measured by a two-probe technique using an electrochemical station (ModuLab MTS Systems). Polymer powders were well-ground into free-flowing fine powders in a mortar by a pestle, followed by drying under vacuum at 100 °C.

The powder (~0.1 g) was quickly mounted to a die (1.6 cm in diameter) and pressed using a hydraulic press equipment at 100 MPa for 1 min. The pellet was then sandwiched between two silver coated block electrodes and sealed in a coin cell (CR 2032). Chronoamperometry (CA) method was used to record the current and calculate ohmic resistance, R. The electronic conductivity was calculated according to the following equations: $\sigma = 1/\rho$, $R = (L/A)\times\rho$, where ρ is resistivity, L is pellet thickness and A is the active membrane area (2.0 cm^2). Pellet thickness was measured by a micrometer.

Electrode fabrication and cell assembly. Electrodes were fabricated via powder dispersion or solution processing. For the powder dispersion method, each polymer powder, Ketjen black and polyvinylidenefluoride (PVDF) binder (100 mg in total) were mixed using a pestle and a mortar with a ratio of 6:3:1 by weight (or 5:4:1 for CV measurements) with ~2 mL NMP added to form a well-dispersed slurry, which was subjected to ultrasonication for 5 minutes and then coated onto an Aluminium foil substrate. For the solution processing method, redox-active PIMs and PVDF binder were fully dissolved in NMP at 60 °C and Ketjen black was added to the polymer solution at a ratio of 6:3:1 by weight (60% of redox-active PIMs). When CNT was used, the weight ratio was 6:3:1:1 for PIM-TMTrip-MHQ, Ketjen black, CNT and PVDF. The mixture was stirred overnight and subjected to ultrasonication for 5 minutes before casting onto an Aluminium foil substrate. The electrodes were dried in an oven at 80 °C for 4 h and at 120 °C under vacuum for 24 h, and then cut into circular electrode discs with a diameter of 10 mm (mass loading: 0.3-0.7 mg cm^{-2}). The electrodes were transferred into a glove box under an Ar atmosphere and assembled into half lithium-ion batteries using CR2032 coin cells. Lithium foil was used as the anode and Celgard 2325 as the separator. To both sides of the cell was added 25 µL electrolyte solution containing 1M lithium bis(trifluoromethanesulfonyl)-imide (LiTFSI) in mixed solvents of 1,3-dioxolane and 1,2-dimethoxyethane (2:1 by Vol.).

Galvanostatic charging/discharging of the batteries was performed within a voltage window of 1.5–3.5 V (versus Li/Li^+) using a Land Battery Tester. Batteries were first activated by discharging, followed by cycling tests at 1 C.

Solid-state Cyclic voltammograms (CVs) were recorded using a Biologic SP-150 potentiostat 1) in a two-electrode coin-cell configuration with 1M LiTFSI DOL/DME as the supporting electrolyte, or 2) in a three-electrode configuration composed of a glassy carbon working electrode, a Pt counter electrode and a Ag/Ag^+ reference electrode (0.01M $AgNO_3$, 0.1M $TBAClO_4$ in acetonitrile) with 0.25M $LiClO_4$, $NaClO_4$ or $TBAClO_4$ in acetonitrile as the

supporting electrolyte. The glassy carbon electrode (diameter: 3mm) was drop-coated with the slurry/solution composed of polymer, Ketjen black and PVDF (6:3:1) followed by drying at 80 °C under vacuum; the active material mass loading was around 0.4 mg cm^{-2}. All electrochemical testing was conducted at 30 °C. coated with the composite of polymer, ketjen black and PVDF (6:3:1 by weight)

4. Computational Methods

Molecular Dynamics (MD) Simulations. MD simulations were conducted using LAMMPS (Large-scale Atomic/Molecular Massively Parallel Simulator)[4] with interactions described by PCFF (polymer consistent forcefield)[5]. Cells of the PIM-TMTrip-MQ and PI-Trip-MQ polymer chains were constructed using the simulated polymerisation algorithm, Polymatic, and 21-step equilibration protocol, previously published by Colina and co-workers[6]. This workflow consists of 5 steps: Step 1: 2 repeat units of each polymer were first optimised at the HF/6-31G* level in Gaussian16[7] to obtain descriptions of both the internal monomer unit and the chain ends. Partial charges were derived by fitting the atomic charges to the optimised structures using the antechamber tool from the Amber package[8]. Partial charges for equivalent atoms were averaged and reassigned by hand. Atom types were assigned using the Forcite module in Materials Studio[9], and reactive atom types labelled where the polymerisation algorithm would later form new bonds. LAMMPS inputs were generated for the internal monomer unit using the msi2lmp tool[4]. Step 2: For each system, 5 initial guess structures were generated by randomly packing 300 monomer units into a periodic box at an arbitrary low density (~0.3 g cm^{-3}). For PIM-TMTrip-MQ, 2 monomer units representing the PMDI and triptycene moieties were packed in a 1:1 ratio. For PI-Trip-MQ, 3 monomer units representing the PMDI moiety and two structural isomers of the triptycene moiety were packed in a 2:1:1 ratio, to mirror the isomeric ratio of the experimental Trip-MQ-diamine precursor. Step 3: Long polymer chains were generated using the distance-based polymerisation algorithm, Polymatic[6], where new bonds were formed between reactive atoms on neighbouring monomers within a cut-off distance of 6 Å. Equal and opposite artificial charges (q_{polym} = ± 0.3e) were placed on reactive atom types during the polymerisation to increase bias towards the bond formation. Energy minimisations were performed between each new bond formation as well as cycles of NVT simulations between every 5 new bond formations to allow for structural rearrangement and to ensure that the system was relaxed throughout. Step 4: Following the polymerisation, any unreacted reactive atom types were saturated with a capping group to mirror the experimental precursors. Specifically, unreacted carbon atoms were capped with an amino

group to mirror the diamine precursor, while unreacted nitrogen atoms were substituted with an oxygen to mirror the PMDA precursor. Any excess charge imposed by the saturation was distributed in small increments across hydrogen atoms. Step 5: Finally, the systems were annealed by the 21-step molecular dynamics equilibration set out by Colina and co-workers, consisting of a cascade of gradual compressions and decompressions at high temperatures and pressures. This equilibration is well established in the literature for the simulation of microporous structures with experimentally comparable densities[6, 10]. The parameters were set as recommended in the literature, where T_{final} = 300 K, T_{max} = 1000 K, P_{final} = 1 bar and P_{max} = 5×10^4 bar[6]. Long-range electrostatic interactions were calculated using the Ewald summation and a Lennard-Jones (LJ) potential was used to describe the short-range interactions with a cut-off distance of 15 Å.

Structural Characterisation. The amorphous PM-TMTrip-MQ and PI-Trip-MQ models were characterised using the Zeo++ package[11] with the high accuracy setting. Reported values for each system are averages of the 5 independent models. Surface areas were measured using a 1.2 Å probe and a 1.55 Å probe with 5000 Monte Carlo (MC) samples. Pore size distributions and visual pore size distributions were measured using a 1 Å probe, with 50,000 and 500,000 MC samples respectively.

Density Functional Theory (DFT) Calculations. DFT calculations were performed using Gaussian16[7], with the Becke's 3 parameter exchange function combined with Lee–Yang–Parr correlation functional (B3LYP)[12, 13] and the 6-31+G(d,p) basis set. Geometry optimisation of a representative hydrogen terminated repeat unit of PIM-TMTrip-MQ was first performed. To predict the lithiation pathway, quantum mechanical (QM) calculations were performed whereby the energies of all possible lithiated isomers of the PIM-TMTrip-MQ-nLi repeat unit were computed. All possible geometries for 1, 2, 3 and 4 lithium additions were explored to reflect the four-electron reversible redox reaction observed experimentally , as well as the 5th and 6th lithium additions that would be required for 4 e^- reduction of the diimide. Backbone carbon and nitrogen atoms were frozen during the optimisations to mirror the rigidity of the long polymer chain. The lithiation pathway was then predicted based on the minimum energy pathway. The Li binding energies ($\Delta E_{binding}$) for each possible lithiation were also predicted, in vacuum, by Equation S1.

$$\Delta E_{binding} = E_{(PIM\text{-}nLi)} - E_{(PIM\text{-}(n\text{-}1)Li)} - E_{(Li)} \qquad \text{(Equation S1)}$$

Where $E_{(PIM\text{-}(n)Li)}$, $E_{(PIM\text{-}(n\text{-}1)Li)}$ and $E_{(Li)}$ represent the DFT calculated energies of the optimised models for a PIM-TMTrip-MQ repeat unit with nLi binding, a PIM-TMTrip-MQ repeat unit with (n-1)Li binding and a Li atom respectively. Visualisations of the optimised geometries and molecular electrostatic potential (MESP) plots were performed using PyMol[14].

To compute the redox potentials of the hydrogen terminated repeat unit, vibrational frequency calculations, with the same levels of theory and basis set, were performed to calculate the Gibbs free energies in the gas phase at different levels of lithiation. Gibbs free energies in solution were also computed using the SMD solvation model for tetrahydrofuran (THF). THF was chosen as the experimentally used solvents were not parameterised for SMD and THF has a similar dielectric perimittivity. The change in Gibbs free energy for each reduction/lithiation step (ΔG_{red}) were approximated by Equation S2.

$$\Delta G_{red} = G_{(PIM\text{-}nLi)} - G_{(PIM\text{-}(n\text{-}1)Li)} - G_{(Li^+)} \qquad \text{(Equation S2)}$$

Where $G_{(PIM\text{-}(n)Li)}$, $G_{(PIM\text{-}(n\text{-}1)Li)}$ and $G_{(Li^+)}$ represent the Gibbs free energies of a PIM-TMTrip-MQ repeat unit with nLi binding, a PIM-TMTrip-MQ repeat unit with (n-1)Li binding and a lithium cation respectively. The reduction potentials for both the gas and solution phases were then computed by equation S3.

$$\Delta E_{red} = \frac{-\Delta G_{red}}{\mathrm{n}F} - 1.4\ \mathrm{V} \qquad \text{(Equation S3)}$$

Where ΔG_{red} is the change in Gibbs free energy upon reduction, n is the number of electrons transferred and F is the Faraday constant. The value of 1.4 V is calculated based on the potential of standard hydrogen electrode (SHE, 4.44 V versus vacuum) and that of the Li/Li^+ electrode (-3.04 V vs SHE). By subtracting 1.4 V here, we convert the theoretical reduction potentials with respect to vacuum to those relative to the Li/Li^+ reference electrode used experimentally.

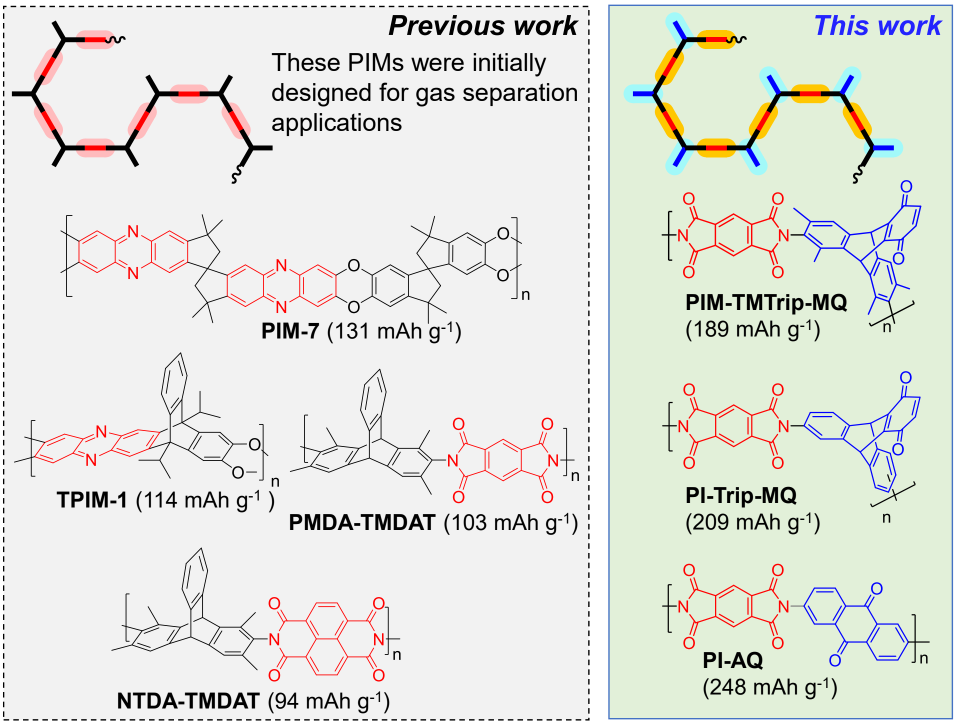

Fig. S2. Summary of redox-active PIMs. PIMs that were initially designed as membrane materials for gas separation may contain redox-active structural units, but their theoretical capacity is too low to be useful.

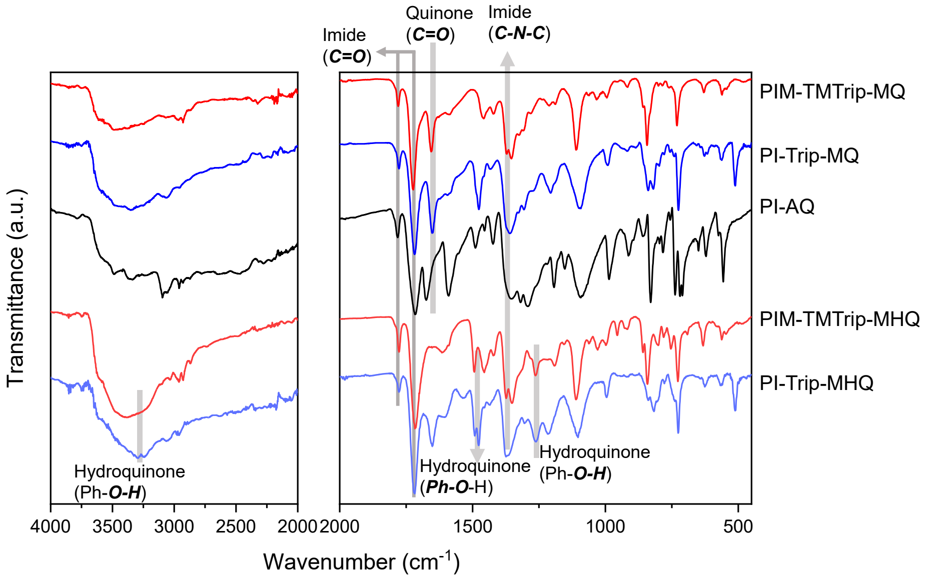

Fig. S3. FTIR of redox-active PIMs.

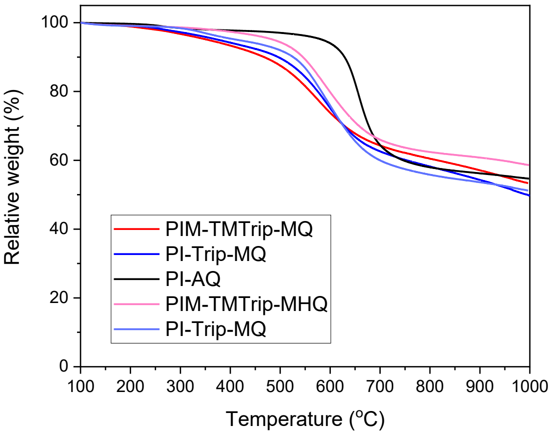

Fig. S4. TGA of redox-active PIMs.

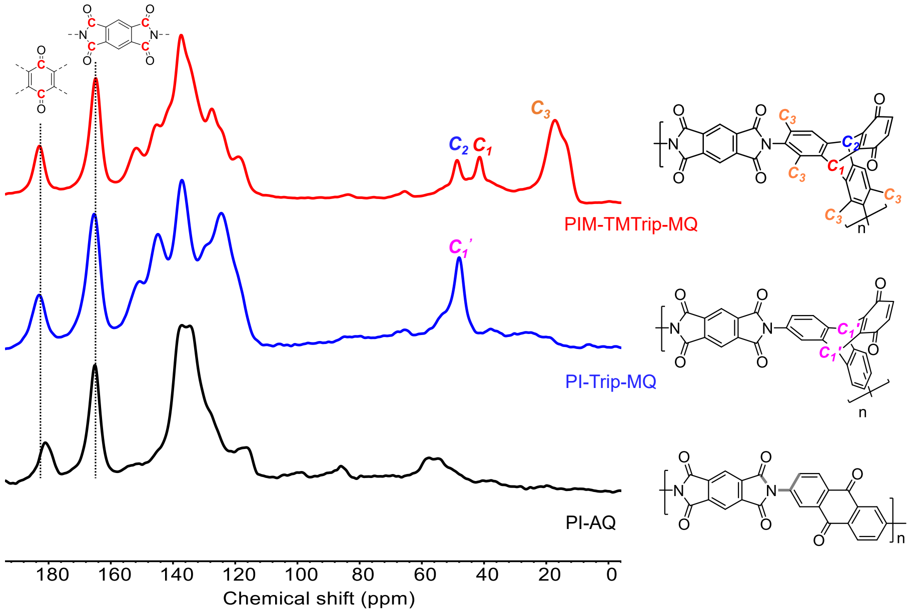

Fig. S5. Solid-state ^{13}C NMR.

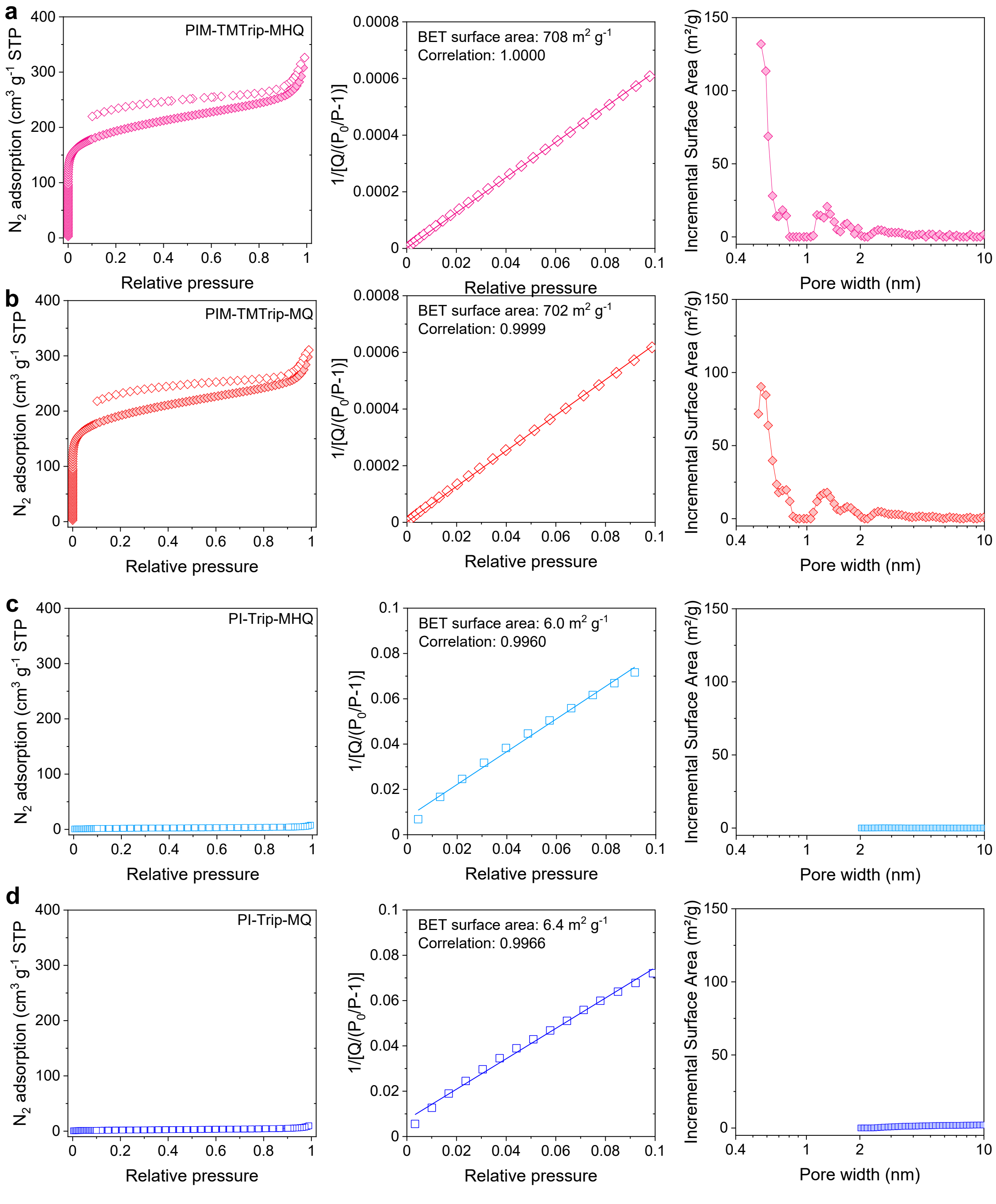

Fig. S6. N_2 sorption at 77K. First column: N_2 sorption isotherm; second column: BET plots; and third column: pore size distribution based on NLDFT calculations.

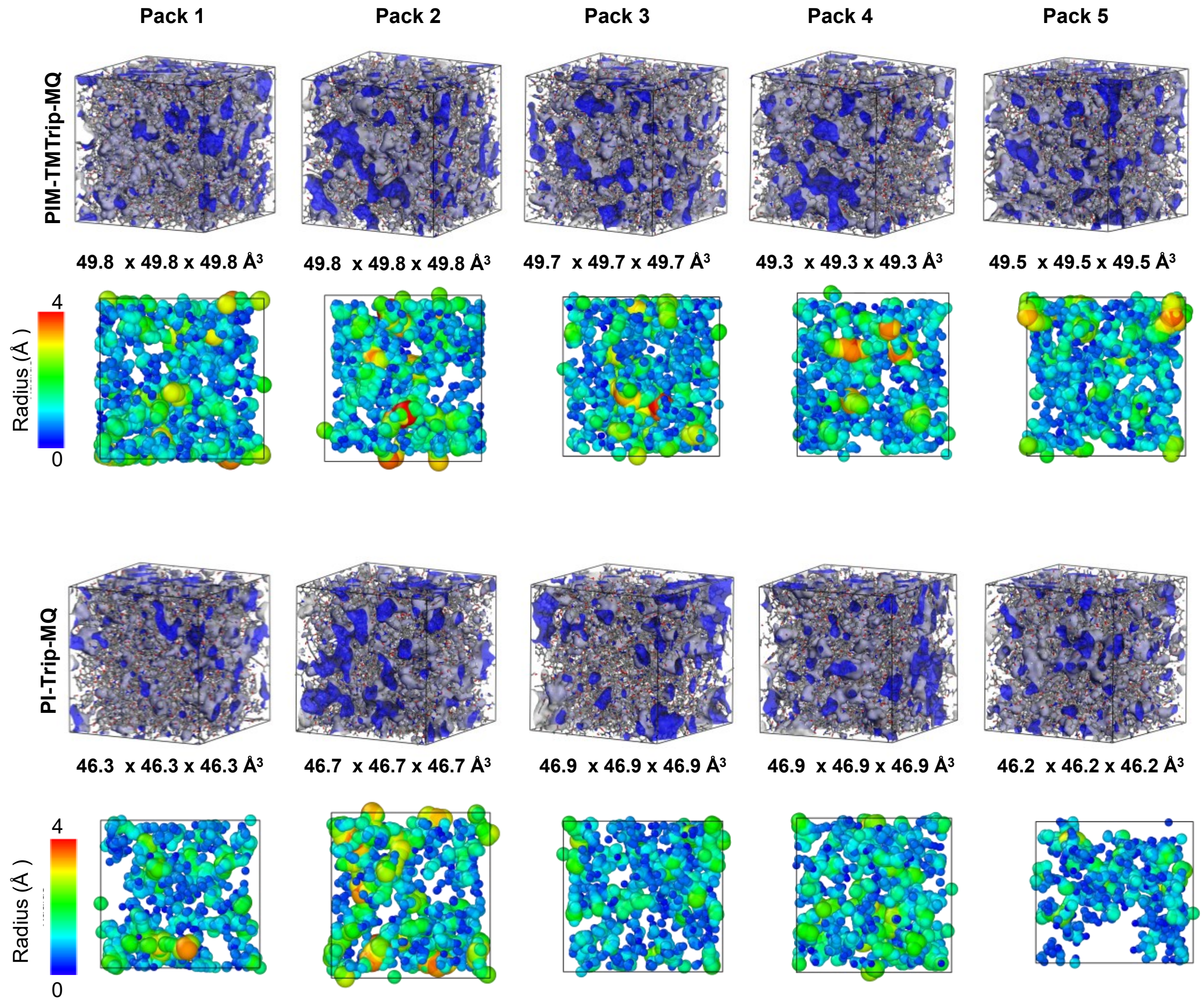

Fig. S7. Amorphous cells of PIM-TMTrip-MQ and PI-Trip-MQ. The surface-mesh highlights the Connolly surface with respect to a probe radius of 1.55 Å (inside and outside are blue and grey respectively). Visual pore size distributions are also shown with each pore color coded by pore radius.

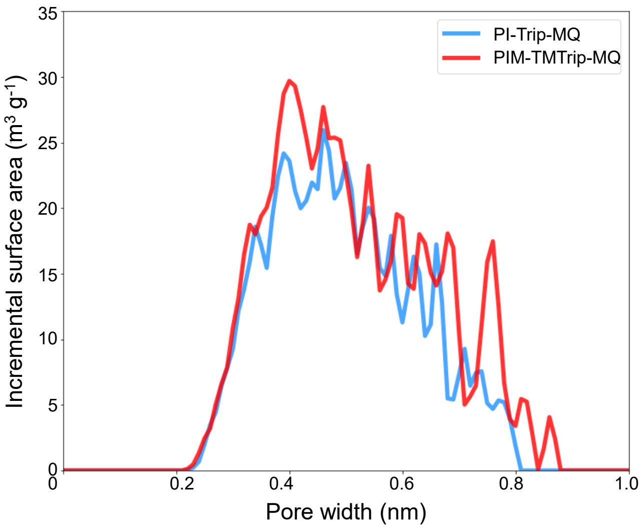

Fig. S8. Simulated pore size distribution (PSD) derived from amorphous cells with respect to a 1 Å probe radius. Good agreement is found between the PSDs of the experimental and simulated systems. The results show that although the two polyimides differ in their magnitudes of measurable surface area, the PSDs of both polyimides encompass a similar landscape. This suggests that whilst the accessibility of voids in PI-Trip-MQ is lesser than those in PIM-TMTrip-MQ, the shapes and sizes of these voids, regardless of accessibility, are comparatively similar in both polymers.

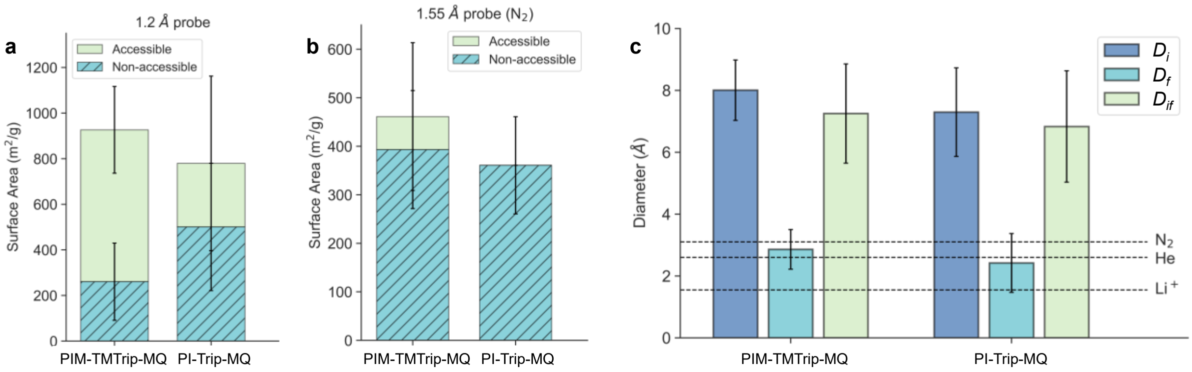

Fig. S9. Geometric surface area analysis of simulated polymer amorphous cells. **a**. **b.** The total geometric surface areas are represented by both accessible and non-accessible surface areas (ASA and NASA respectively). Describing surface area in this way provides deeper insight into the nature of the microporous network; ASA is representative of the interconnected free volume throughout the network, while NASA defines any isolated voids which could become accessible if host-guest interactions and dynamic flexibility of the real systems were considered (for example, swelling). While the absolute balance between NASA and ASA should not be overanalysed in a rigid model, they provide qualitative insight into the different systems' relative porosity levels. **c**. Measurements of the largest included sphere (D_i), the largest free sphere path (D_f), and the largest included sphere along the free sphere path (D_{if}) for the polymers and comparison with the diameters of some common sorbents as well as the lithium cation. Notably, D_f defines the largest probe diameter which can diffuse throughout the model through interconnected pores, analogous to the pore limiting diameter. D_f values for PIM-TMTrip-MQ and PI-Trip-MQ (2.86 Å and 2.42 Å respectively) show that, although regions of free volume are present In both polyimides, these regions are less accessible to guest molecules in the latter. This is an example of how tuneable selectivity can be achieved in PIMs by functionalisation. We can surmise that, because of the enhanced rigidity in PIM-TMTrip-MQ, inefficient packing of the polyimide chains results in larger and more accessible pores. This is a particularly important feature in battery applications, where enhancing the accessibility of redox-active sites can lead to faster diffusion kinetics.

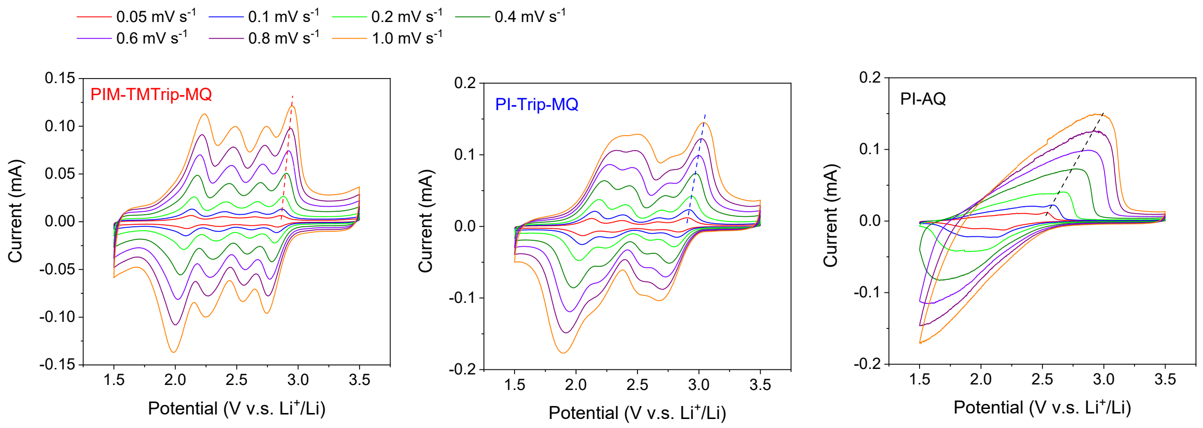

Fig. S10. Solid-state CV at varied scanning rates. All three polymers undergo reversible oxidation and reduction via a four-electron per repeat unit process. While the microporous PIM-TMTrip-MQ and PI-Trip-MQ showed well-resolved peaks, PI-AQ exhibited broad and overlapping peaks across the scanning rates. The anodic peak with the highest potential (~ 2.9 V vs. Li^+/Li) was used to derive the log-log plot of peak current versus scanning rate (Fig 3c), as this anodic peak showed the least overlap with other peaks for all three polymers. Electrolyte: 1M LiTFSI in DOL/DME

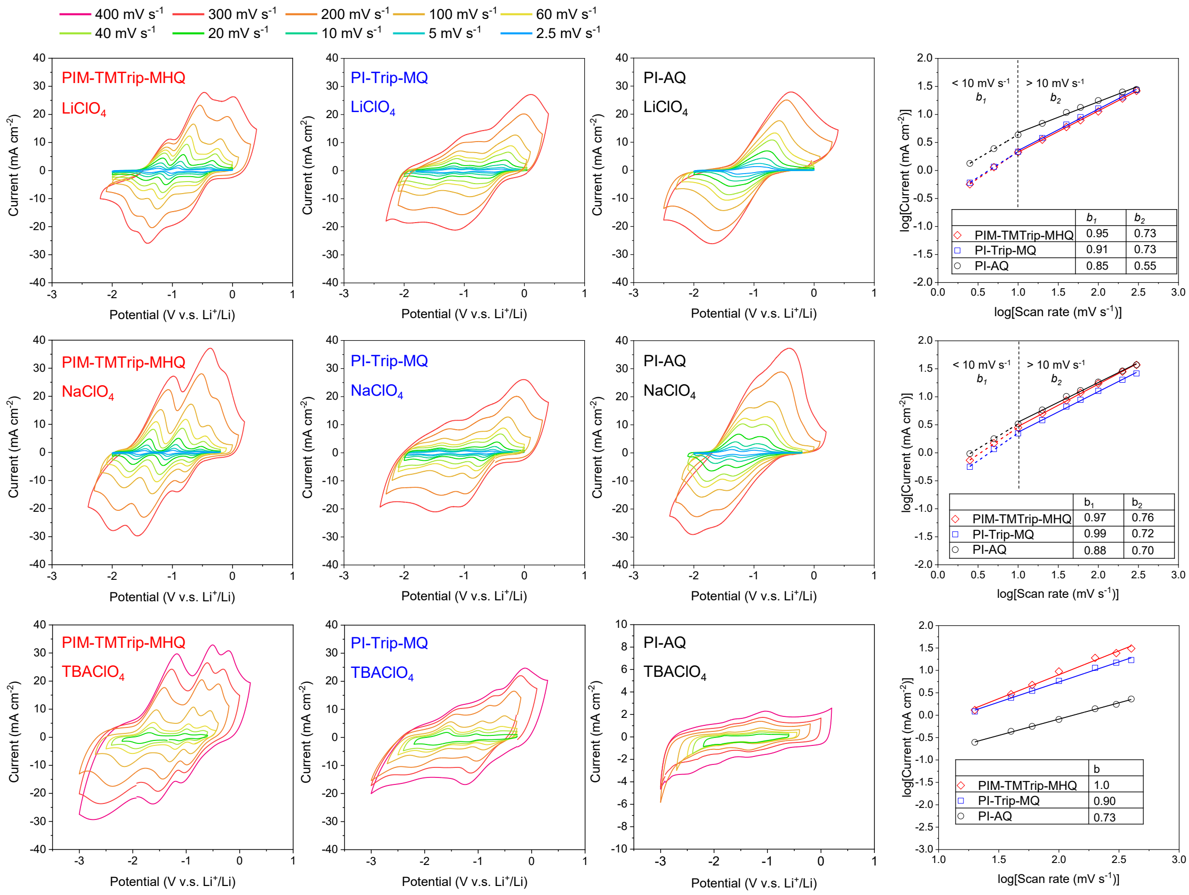

Fig. S11. Solid-state CV in acetonitrile electrolyte solutions. To link the micropore size of redox-active PIMs with the size of counterions, we chose $LiClO_4$, $NaClO_4$, and $TBAClO_4$ as the supporting electrolyte. The highest *b* values were found for the most porous PIM-TMTrip-MHQ while the non-porous PI-AQ showed the lowest *b* values, which is consistent with the observation when LiTFSI was used. It should be noted that we observed partial peeling-off of the coated composite electrode of PI-AQ from the working electrode in $TBAClO_4$ electrolyte, which led to its lower peak intensity.

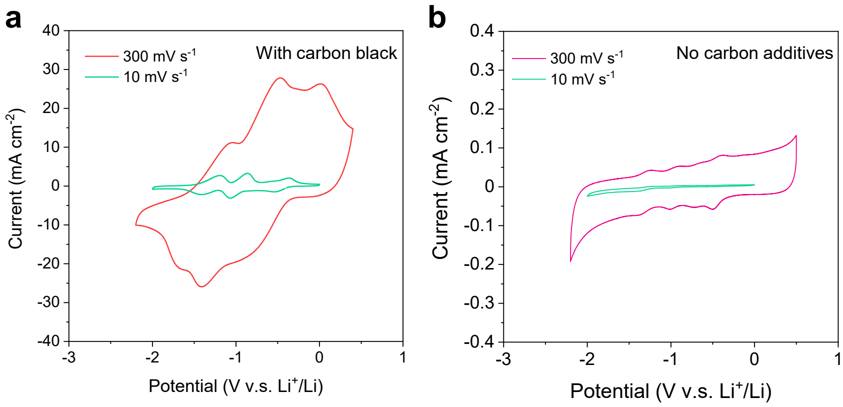

Fig. S12. Solid-state CV of electrodes with or without carbon additives. Electrolyte: $LiClO_4$ in acetonitrile. Electrode composition: **a**. PIM-TMTrip-MHQ, KJB and PVDF in a weight ratio of 6:3:1, b. PIM-TMTrip-MHQ and PVDF in a weight ratio of 6:1

**PIM-TMTrip-MQ**	**Lithium atom**		
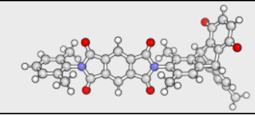			
E = -59311.09 eV	E = -203.84 eV		
**PIM-TMTrip-MQ-1Li**			
Case 1	***Case 2***	Case 3	Case 4
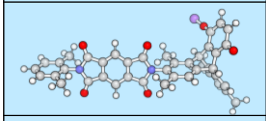	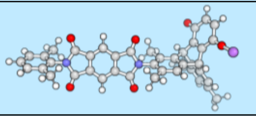	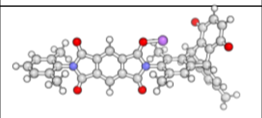	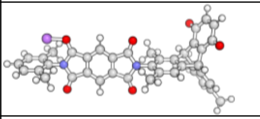
E = -59517.28 eV	***E = -59517.28 eV***	*E* = -59517.06 eV	*E* = -59516.91 eV
BE = -2.35 eV	***BE = -2.35 eV***	*BE* = -2.13 eV	*BE* = -1.98 eV
Case 5	Case 6		
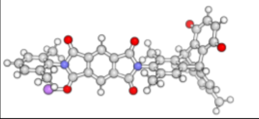	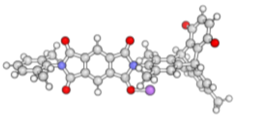		
E = -59516.91 eV	*E* = -59516.99 eV		
BE = -1.98 eV	*BE* = -2.06 eV		
**PIM-TMTrip-MQ-2Li**			
Case 1	Case 2	Case 3	Case 4
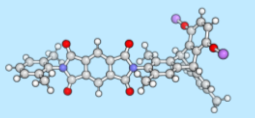	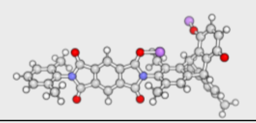	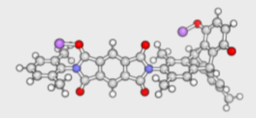	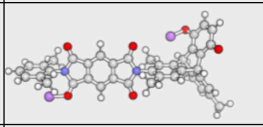
E = -59723.38 eV	*E* = -59722.68 eV	*E* = -59722.37 eV	*E* = -59722.38 eV
BE = -2.26 eV	*BE* = -1.56 Ev	*BE* = -1.25 eV	*BE* = -1.26 eV
Case 5			
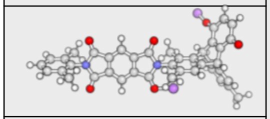			
E = -59722.60 eV			
BE = -1.48 eV			
**PIM-TMTrip-MQ-3Li**			
Case 1	Case 2	Case 3	Case 4
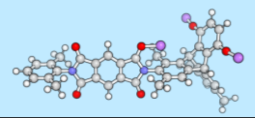	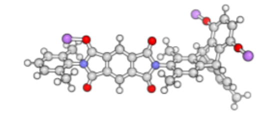	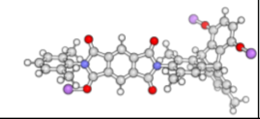	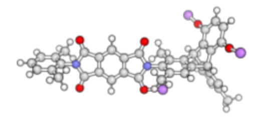
E = -59929.52 eV	*E* = -59929.10 eV	*E* = -59929.11 eV	*E* = -59929.43 eV
BE = -2.30 eV	*BE* = -1.88 eV	*BE* = -1.88 eV	*BE* = -2.20 eV
**PIM-TMTrip-MQ-4Li**			
Case 1	***Case 2***	Case 3	
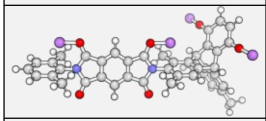	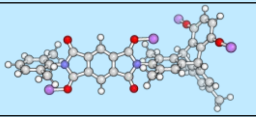	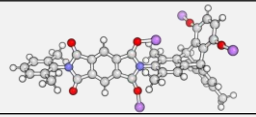	
E = -60134.75 eV	***E = -60134.85 eV***	*E* = -60134.72 eV	
BE = -1.39 eV	***BE = -1.48 eV***	*BE* = -1.36 eV	
**PIM-TMTrip-MQ-5Li**	**PIM-TMTrip-MQ-6Li**		
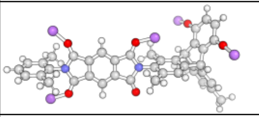	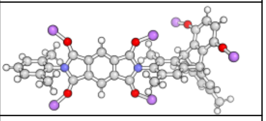		
E = -60339.92 eV	*E* = -60544.51 eV		
BE = -1.23 eV	*BE* = -0.75 eV		

Figure S13. Prediction of lithiation pathways based on the minimum energy principle. All possible Li-binding geometries for the PIM-TMTrip-MQ-nLi (n=0, 1, 2, 3 and 4) isomers based on DFT calculations. Although they weren't included in our conformational search, optimised geometries for n=5 and n=6 repeat units are also included, as well as the energy of a lithium atom which was included in the computed binding energies. *E* denotes the DFT calculated energy for each geometry and *BE* denotes the binding energy for each possible lithiation step with respect to the preceding lowest energy lithiated isomer. Constitutiional isomers with the lowest total energy and strongest binding energy are predicted to be the preferred lithiation sites (highlighted in red and blue). The DFT calculations suggest that the first and second lithiation would take place on the triptycene carbonyls, whilst the third and fourth lithiation would take place on the PMDA unit. Atoms in grey, white, red, blue, and violet represent carbon, hydrogen, oxygen, nitrogen, and lithium, respectively.

Table S1. Redox potential calculations. Computed Gibbs free energies (*G*) for the PIM-TMTripMQ-nLi (n=0, 1, 2, 3, 4, 5 and 6) isomers and a lithium cation are reported in the gas and solution phases. Gibbs free energy changes (ΔG_{red}) and predicted redox potentials (ΔE_{red}) during each possible reduction are reported for both the gas and solution phases. Experimental redox potentials are also recorded for comparison.

	**Gas Phase**			**Solution (SMD, THF)**			**Experimental**
	G (eV)	ΔG_{red} (eV)	ΔE_{red} (V, Li/Li$^+$)	G (eV)	ΔG_{red} (eV)	ΔE_{red} (V, Li/Li$^+$)	ΔE_{red} (V, Li/Li$^+$)
Li^+	-198.57	-	-	-201.95	-	-	-
PIM-TMTrip-MQ	-59301.90	-	-	-59303.48	-	-	-
PIM-TMTrip-MQ-1Li	-59508.16	-7.70	6.30	-59510.35	-4.91	3.52	2.84
PIM-TMTrip-MQ-2Li	-59714.27	-7.54	6.14	-59716.81	-4.51	3.11	2.64
PIM-TMTrip-MQ-3Li	-59920.43	-7.59	6.19	-59923.31	-4.54	3.14	2.37
PIM-TMTrip-MQ-4Li	-60125.74	-6.74	5.34	-60129.12	-3.85	2.45	2.11
PIM-TMTrip-MQ-5Li	-60330.91	-6.60	5.20	-60333.91	-2.84	1.44	-
PIM-TMTrip-MQ-6Li	-60535.57	-6.09	4.69	-60538.71	-2.84	1.44	-

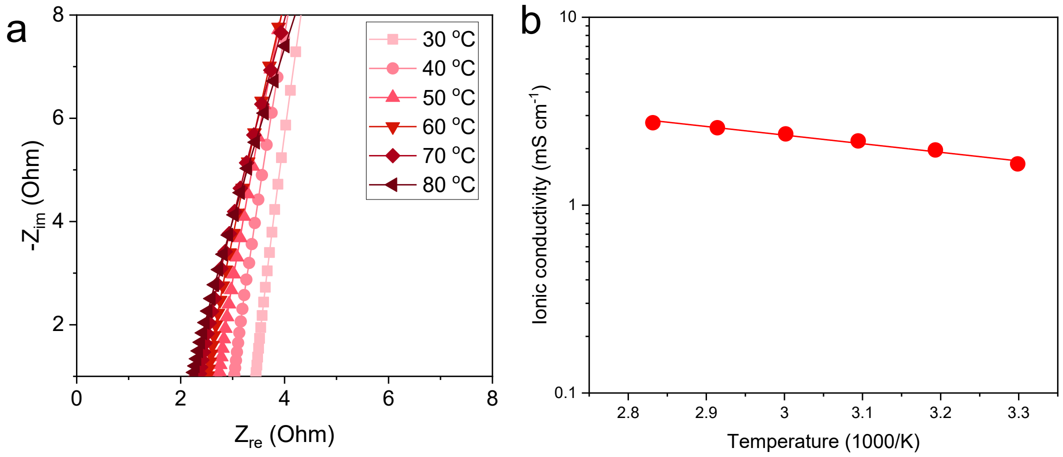

Fig. S14. Ionic conductivity of a solution-cast PIM-TMTrip-MHQ film. Film thickness: 112 μm. The polymer film was infiltrated with 1M LiTFSI in DOL/DME (2:1) by soaking in the electrolyte overnight at room temperature prior to assembly in coin cell hardware.

Table S2. Single gas permeability and ideal selectivity of self-supported PIM-TMTrip-MHQ film.

Ageing time	Single gas permeability (Barrer)						Ideal selectivity		
	He	H_2	O_2	CO_2	N_2	CH_4	CO_2/CH_4	H_2/N_2	O_2/N_2
Fresh	970	2422	565	3146	116	104	30	21	4.9
4 days	819	1980	460	2775	95	81	34	21	4.9

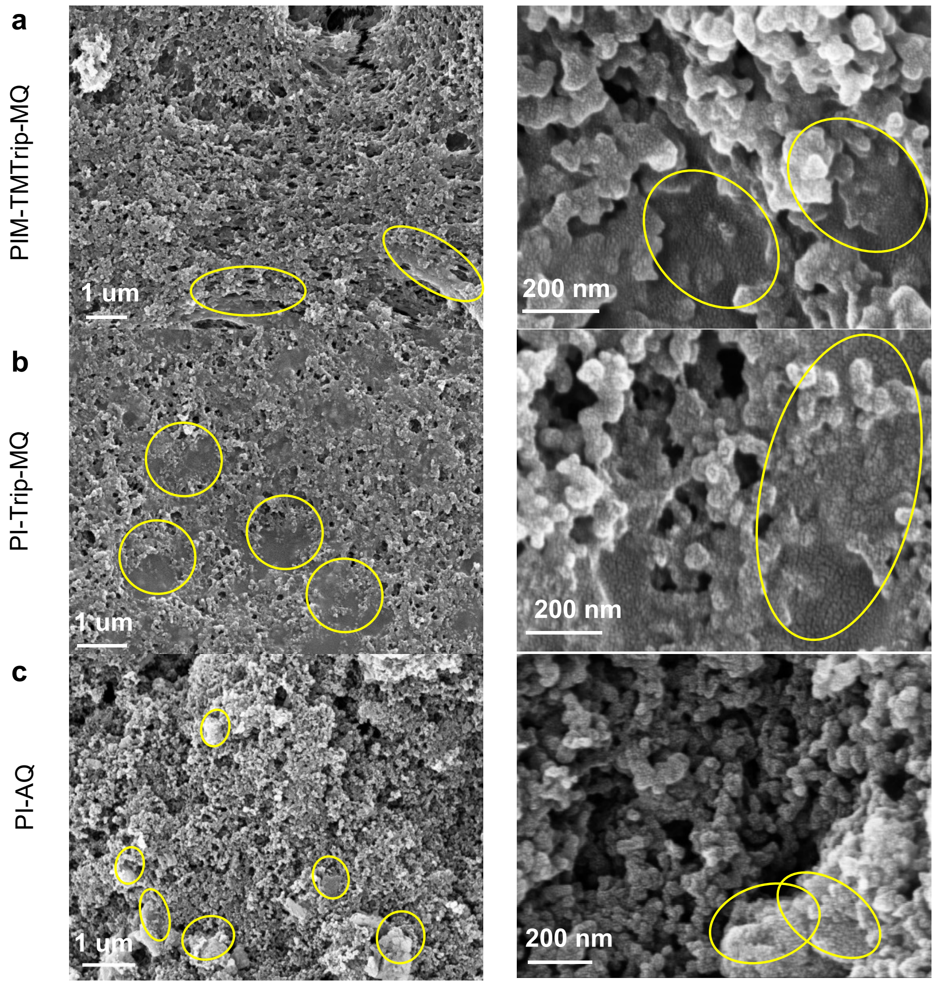

Fig. S15. SEM images of the fresh electrode. Fabrication method: powder dispersion. Large polymer flakes and particles are highlighted by yellow circles.

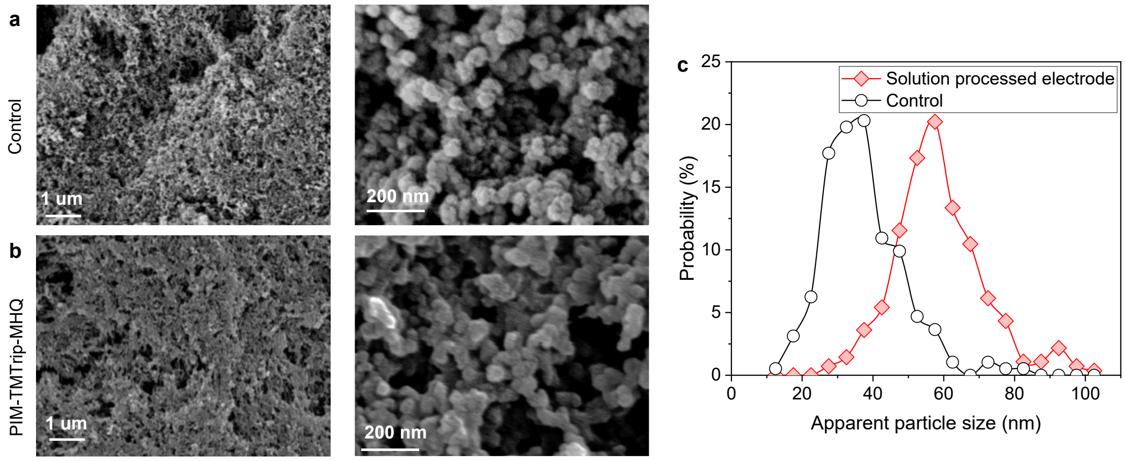

Fig. S16. SEM images of the fresh electrode. Fabrication method: solution processing. Particle size was measured using ImageJ. The control sample is composed of carbon black and PVDF binder (3:1 by weight), while the solution-processed electrode is composed of PIM-TMTrip-MHQ, carbon black and PVDF binder (6:3:1 by weight). It should be noted the apparent particle size measured from SEM includes the intrinsic size of the particles and also that of the conductive coating layer that was sputtered prior to SEM tests to avoid sample charging.

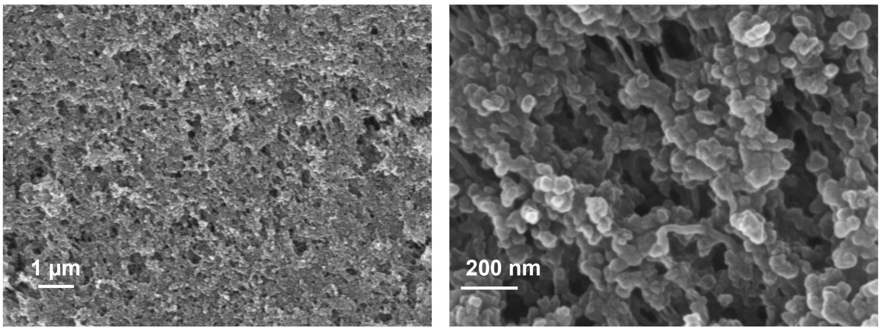

Fig. S17. SEM images of the fresh electrode. Fabrication method: solution processing. The sample is composed of PIM-TMTrip-MHQ, Ketjen black, CNT and PVDF binder (6:3:1:1 by weight).

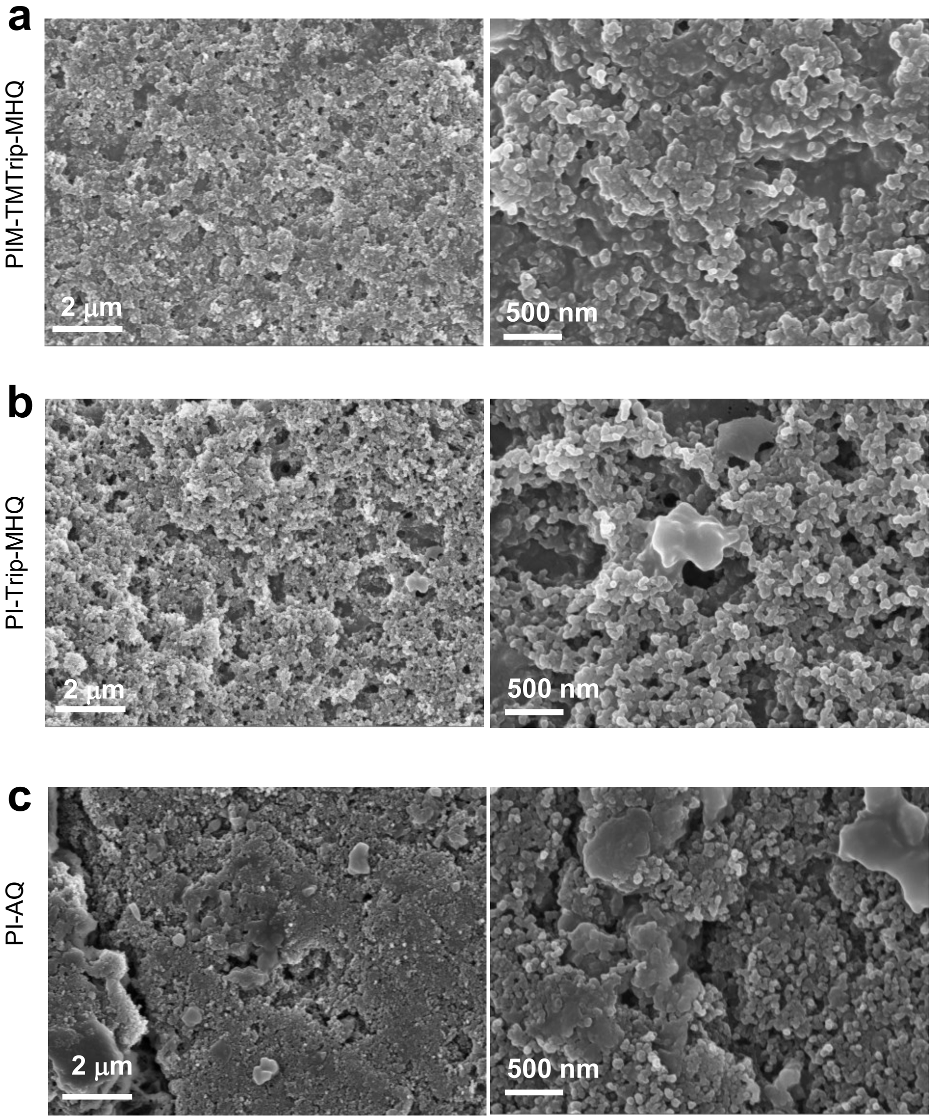

Fig. S18. SEM images of the post-cycling electrodes.

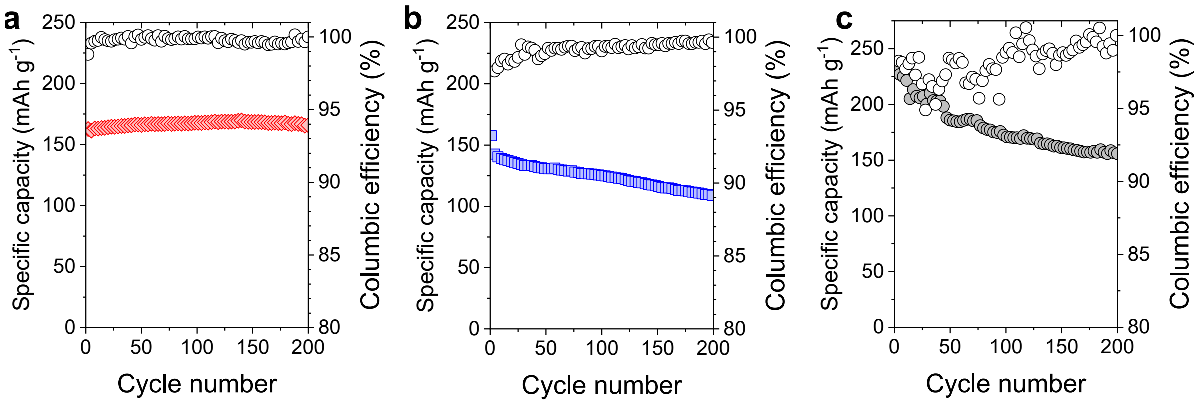

Fig. S19. Cycling performance. a. PIM-Trip-MHQ, **b.** PI-Trip-MQ, and **C.** PI-AQ. The discharge capacity is normalized and presented in Figure 5e.

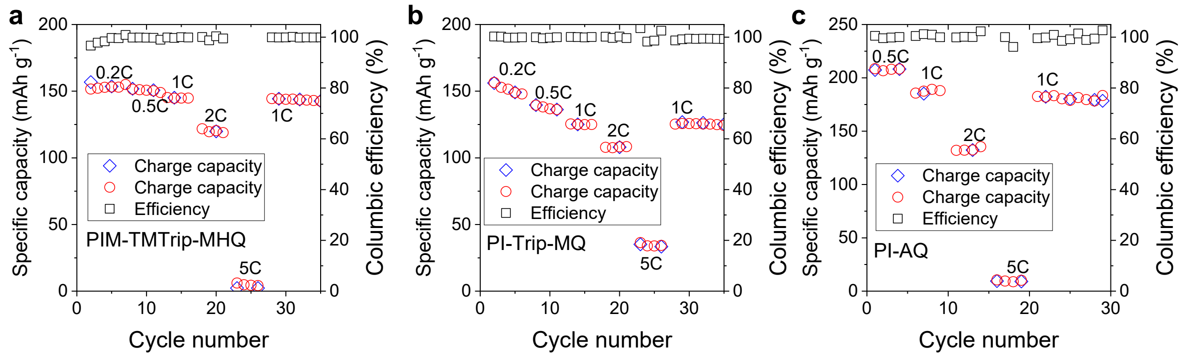

Fig. S20. Rating performance. Electron transfer is the limiting step in the rating performance due to the poor electronic conductivity of these non-conjugated redox PIMs.

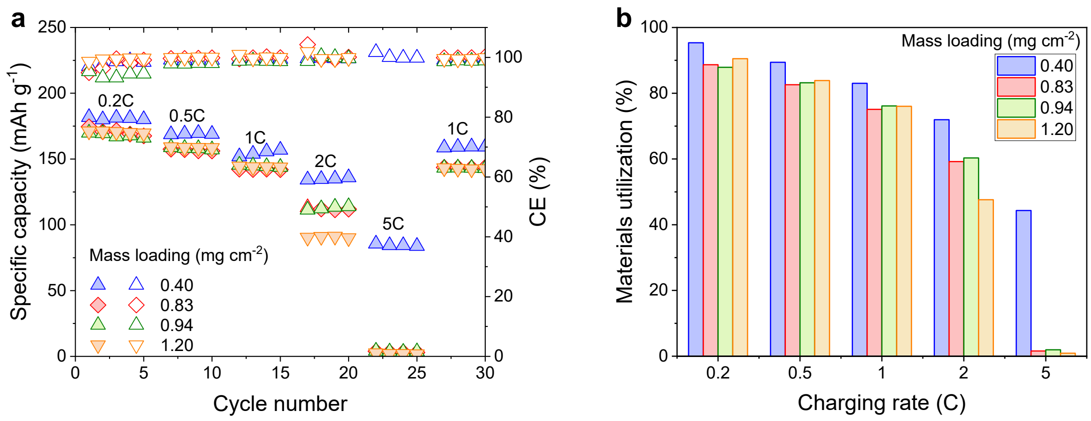

Fig. S21. Rate performance at varied mass loading. Electrode composition: PIM-TMTrip-MHQ, KJB, CNT and PVDF binder in a weight ratio of 6:3:1:1. Values shown in the figures are based on the mass loading of active materials.

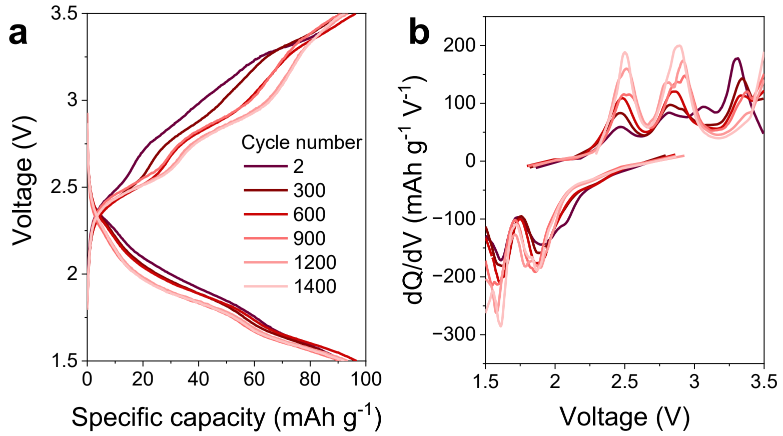

Fig. S22. Incremental capacity analysis. a. charging-discharging profiles. **b**. incremental capacity as a function of voltage. Full cycling is shown in Fig. 6.

References

1. Song, Q. et al. Zeolitic imidazolate framework (ZIF-8) based polymer nanocomposite membranes for gas separation. *Energy & Environmental Science* **5**, 8359-8369 (2012).
2. Ghanem, B.S., Alghunaimi, F., Wang, Y., Genduso, G. & Pinnau, I. Synthesis of Highly Gas-Permeable Polyimides of Intrinsic Microporosity Derived from 1,3,6,8-Tetramethyl-2,7-diaminotriptycene. *ACS Omega* **3**, 11874-11882 (2018).
3. Chen, X. et al. Integrating single Ni sites into biomimetic networks of covalent organic frameworks for selective photoreduction of CO 2. *Chemical science* **11**, 6915-6922 (2020).
4. Plimpton, S., Crozier, P. & Thompson, A. LAMMPS-large-scale atomic/molecular massively parallel simulator. *Sandia National Laboratories* **18**, 43 (2007).
5. Sun, H. Force field for computation of conformational energies, structures, and vibrational frequencies of aromatic polyesters. *Journal of Computational Chemistry* **15**, 752-768 (1994).
6. Abbott, L.J., Hart, K.E. & Colina, C.M. Polymatic: a generalized simulated polymerization algorithm for amorphous polymers. *Theoretical Chemistry Accounts* **132**, 1-19 (2013).
7. Frisch, M. et al. Gaussian 16, Gaussian. *Inc., Wallingford CT* **2016** (2016).
8. Wang, J., Wang, W., Kollman, P.A. & Case, D.A. Antechamber: an accessory software package for molecular mechanical calculations. *J. Am. Chem. Soc* **222**, U403 (2001).
9. Inc, A. (2017).
10. Hart, K.E., Abbott, L.J., McKeown, N.B. & Colina, C.M. Toward effective CO2/CH4 separations by sulfur-containing PIMs via predictive molecular simulations. *Macromolecules* **46**, 5371-5380 (2013).
11. Willems, T.F., Rycroft, C.H., Kazi, M., Meza, J.C. & Haranczyk, M. Algorithms and tools for high-throughput geometry-based analysis of crystalline porous materials. *Microporous and Mesoporous Materials* **149**, 134-141 (2012).
12. Becke, A.D. Density-functional exchange-energy approximation with correct asymptotic behavior. *Physical review A* **38**, 3098 (1988).
13. Lee, C., Yang, W. & Parr, R.G. Development of the Colle-Salvetti correlation-energy formula into a functional of the electron density. *Physical review B* **37**, 785 (1988).
14. Schrödinger, L. The PyMOL Molecular Graphics System, Version 2.0 Schrödinger, LLC (2017).
